# Metabolic engineering to simultaneously activate anthocyanin and proanthocyanidin biosynthetic pathways in *Nicotiana spp*.

**DOI:** 10.1371/journal.pone.0184839

**Published:** 2017-09-13

**Authors:** Sandra Fresquet-Corrales, Edelín Roque, Alejandro Sarrión-Perdigones, Maricruz Rochina, María P. López-Gresa, Huertas M. Díaz-Mula, José M. Bellés, Francisco Tomás-Barberán, José P. Beltrán, Luis A. Cañas

**Affiliations:** 1 Instituto de Biología Molecular y Celular de Plantas (CSIC-UPV), Valencia, Spain; 2 Department of Biochemistry and Molecular Biology, Baylor College of Medicine, Houston, Texas, United States of America; 3 Centro de Edafología y Biología Aplicada del Segura (CEBAS-CSIC), Research Group on Quality, Safety and Bioactivity of Plant Foods, Murcia, Spain; Zhejiang University, CHINA

## Abstract

Proanthocyanidins (PAs), or condensed tannins, are powerful antioxidants that remove harmful free oxygen radicals from cells. To engineer the anthocyanin and proanthocyanidin biosynthetic pathways to *de novo* produce PAs in two *Nicotiana* species, we incorporated four transgenes to the plant chassis. We opted to perform a simultaneous transformation of the genes linked in a multigenic construct rather than classical breeding or retransformation approaches. We generated a GoldenBraid 2.0 multigenic construct containing two *Antirrhinum majus* transcription factors (*AmRosea1* and *AmDelila*) to upregulate the anthocyanin pathway in combination with two *Medicago truncatula* genes (*MtLAR* and *MtANR*) to produce the enzymes that will derivate the biosynthetic pathway to PAs production. Transient and stable transformation of *Nicotiana benthamiana* and *Nicotiana tabacum* with the multigenic construct were respectively performed. Transient expression experiments in *N*. *benthamiana* showed the activation of the anthocyanin pathway producing a purple color in the agroinfiltrated leaves and also the effective production of 208.5 nmol (-) catechin/g FW and 228.5 nmol (-) epicatechin/g FW measured by the *p*-dimethylaminocinnamaldehyde (DMACA) method. The integration capacity of the four transgenes, their respective expression levels and their heritability in the second generation were analyzed in stably transformed *N*. *tabacum* plants. DMACA and phoroglucinolysis/HPLC-MS analyses corroborated the activation of both pathways and the effective production of PAs in T0 and T1 transgenic tobacco plants up to a maximum of 3.48 mg/g DW. The possible biotechnological applications of the GB2.0 multigenic approach in forage legumes to produce “bloat-safe” plants and to improve the efficiency of conversion of plant protein into animal protein (ruminal protein bypass) are discussed.

## Introduction

The colorless flavonoid polymers called proanthocyanidins (PAs) or condensed tannins (CTs), and their monomeric building blocks, the (epi)-flavan-3-ols (+) catechin and (-)-epicatechin, are important bioactive compounds both for human health and agriculture, and their production in crop plants using metabolic engineering would permit the design of à-la-carte nutraceutical foods. Oligomeric and polymeric PAs are end products of the flavonoid biosynthetic pathway. Flavonoids constitute one of the largest groups of plant secondary metabolites derived from the phenylpropanoid and acetate/malonate metabolic pathways [1–3]. Several types of transcription factors (TFs), including basic helix-loop-helix (bHLH), MYB and WD-40, control the expression of genes coding for enzymes of this pathway [4]. These TFs act together to regulate the expression of genes encoding enzymes such as chalcone synthase (CHS), chalcone isomerase (CHI), flavanone 3-hydroxylase (F3H), dihydroflavonol 4-reductase (DFR), anthocyanidin synthase (ANS) and UDP glucose-flavonoid 3-O-glucosyltransferase (UFGT), [5–12]. One approach to genetically modified plants to produce PAs is to transform first plants with a transcription factor or factors which presence increase the availability of upstream precursors of PAs therefore inducing anthocyanidin production [13]. Then, in a second step the co-expression of one or more genes coding for PA-specific biosynthetic enzymes might lead to PAs accumulation. For example, the conversion of cyanidin to the flavan-3-ol (-)-epicatechin, a building block of PAs, has been accomplished by such a strategy [1, 14].

The anthocyanin branch of flavonoid biosynthesis is activated in *Antirrhinum majus* by two TFs, *AmRosea1* (encoding a MYB-related transcription factor) and *AmDelila* (encoding a bHLH transcription factor), [15, 16]. These TFs have been used to produce high levels of anthocyanin in the tomato fruit by their expression under the control of the fruit-specific E8 promoter [17]. Once the biosynthesis of anthocyanins is activated in a plant tissue to direct the flavonoid pathway to PAs production, the activity of leucoanthocyanidin reductase (LAR) to produce (+)-catechin and (+)-gallocatechin and anthocyanidin reductase (ANR), to produce (-)-epicatechin and (-)-epigallocatechin are necessary [18–21].

In the last decades, technical hurdles limiting the number of genes transferred to plants have introduced a significant bottleneck to progress in plant biotechnology [22–24]. The genes are stacked in transgenic plants using iterative processes, such as successive rounds of crosses between different transgenic lines [25, 26], or the sequential transformation of transgenic plants with additional transgenes [27–29]. Both approaches have two major drawbacks: the long and labor-intensive processes involving several breeding generations and the fact that the different transgenes are unlinked, leading to segregation in subsequent generations [30]. The alternative to unlinked transformation is the simultaneous transfer to the plant of several genetic units assembled into the same DNA string (Multigene transfer; MGT). This approach facilitates not only the inheritance of all the transgenes among generations but also the selection of a successful transformation event, because only one selection marker is required. Several methods have been implemented to facilitate the assembly of more than two genes in the same plasmid but most of them are limited in the number of parts to be assembled (Multisite Gateway) [31, 32], and others require *de novo* synthesis of parts in each assembly reaction to add overlapping regions limiting the standardization and reusability [33]. Therefore, genetic engineering has progressively evolved from single-gene intervention to multigene transformation to tackle increasingly ambitious objectives [23]. An alternative approach is the modular construction of genetic devices using standardized DNA parts. Modular design facilitates combinatorial engineering, as standard DNA parts can be easily exchanged improving the possibilities of the building process. A good example for this is the GoldenBraid 2.0 (GB2.0) cloning system a standardized DNA assembly platform developed to facilitate multigene engineering in plants by the simultaneous incorporation of several transgenes (https://gbcloning.upv.es/). GB2.0 allows the binary combination of multipartite assemblies using an extremely simple set of rules. This cloning system facilitates the reusability of DNA parts and assembled devices to efficiently build complex constructs and includes a collection of pre-assembled genetic devices that facilitate the generation of multigenic constructs [34–36]. This multigenic cloning system has been proved by transient expression (agroinfiltration) in *Nicotiana benthamiana* leaves and in stable transformation of *N*. *benthamiana* and *Nicotiana tabacum* [37, 38].

An interesting challenge in plant metabolic engineering is the introduction of PAs into alfalfa (*Medicago sativa* L.) forage, which would be particularly important to ruminant livestock producers to combat “pasture bloat”, a digestive pathology caused by the production of greenhouse gases in the rumen due to excessive fermentation of dietary protein from forages [2]. Currently, the livestock diet should be complemented with surfactants to break down the protein foams or mixed with forage known to contain moderate levels of PAs, unfortunately both are costly options. The presence of PAs into alfalfa could help to fight pasture bloat and improve the efficiency of conversion of plant protein into animal protein (ruminal protein bypass). The lack of PAs in the leaves of the major forage legume such as alfalfa has prompted studies for the understanding of the molecular and cellular biology of PA polymerization, transport and storage helped by the functional genomics tools available in *Medicago truncatula* [3, 4, 20, 21].

Here we report the engineering of the flavonoid biosynthetic pathway to produce the monomeric building blocks of PAs by multigene transfer using the GoldenBraid2.0 (GB2.0) cloning system. The GB2.0 construct was designed to carry two *A*. *majus* sequences coding for TFs (*AmRosea1* and *AmDelila*) that activate the anthocyanin biosynthetic pathway and two sequences coding for the PA-specific biosynthetic enzymes (*MtANR* and *MtLAR*) from *M*. *truncatula*. Transient expression experiments in *N*. *benthamiana* and stable transformation of *N*. *tabacum* showed the activation of both pathways and the production of monomeric components of PAs (+) catechin and (-) epichatechin in the transgenic plants, therefore supporting the use of the GoldenBraid 2.0 cloning system to modify this metabolic pathway in plant species.

## Materials and methods

### Plant material

Plants of *N*. *benthamiana* were grown from seeds in a greenhouse at 24°C day/ 20°C night in a 16 h light/8 h dark cycle. *M*. *truncatula* 2HA plants were grown in a growth chamber with 16 h light/8 h dark photoperiod and temperature range of 22°C day/18°C night. Tobacco plants (*N*. *tabacum* cv Petite Havana SR1) were grown in a mixture (1:1) of sphagnum:vermiculite. Plants were irrigated with Hoagland N°.1 solution supplemented with oligoelements [39] and cultivated under 16 h photoperiods in a greenhouse at 25°C day/18°C night. Supplementary lighting was provided by 400 W Phillips HDK/400 HPI [R] [N].

### GoldenBraid 2.0 reactions

Restriction-ligation reactions were performed as described previously [35, 40]. Briefly, 75 ng of the destination vectors and the parts/modules to be assembled were mixed with 1μL of T4 Ligase (Promega) and the appropriate Type IIs restriction enzyme (*i*.*e*., *Bsa*I for the alfa assemblies and *Bsm*BI for the omega assemblies and the domestication of GBparts) in a final volume of 10 μL. These reaction mixes were generally incubated for 25 Golden Gate cycles (2 min at 37°C, 5 min at 16°C). One microliter of each reaction was transformed into *Escherichia coli* DH5α electrocompetent cells. Positive clones were selected on LB agar plates supplemented with appropriated antibiotics. For the GBparts domestication reactions, ampicillin at 100 μg mL^**-1**^, 0.5 mM IPTG and X-Gal (20 mg/ml) were used. For Alfa and Omega assemblies we used kanamycin and spectinomycin at 50 μg mL^**-1**^, respectively. The binary vectors generated by the GB2.0 multigenic approach were transformed into *Agrobacterium tumefaciens* strains GV3101 and LBA4404, which were used for plant transformation experiments. Plasmid DNA was extracted using the E.Z.N.A. Plasmid Mini Kit I (Omega Bio-Tek, Norcross, GA, USA). Correct assemblies were confirmed by restriction analysis and sequencing when appropriate.

### Domestication of the *MtANR* and *MtLAR* GBparts

The coding sequences of *MtANR* and *MtLAR* were adapted to the GB2.0 grammar as described earlier [35]. This process is known as domestication, and involves the addition of the appropriate flanking overhangs to the DNA parts, and also the occasional removal of internal Type IIs restriction sites by introducing silent mismatches to disrupt the enzyme target sites. *MtANR* and *MtLAR* were isolated by RT-PCR from RNA of *M*. *truncatula* seeds 10 days after pollination using specific primers designed from the available sequences (NCBI: XM_013601695.1 and XM_003591782 respectively). Both *MtANR* and *MtLAR* coding sequences included internal restriction sites that had to be removed. *MtANR* was amplified using the primers MtANR-F1 and MtANR-R1 for the first fragment, and MtANR-F2 and MtANR-R2 for the second one (S1 Table). *MtLAR* was amplified using the primers MtLAR-F1 and MtLAR-R1 for the first fragment, and MtLAR-F2 and MtLAR-R2 for the second one (S1 Table). Amplified bands were purified using the QIAquick PCR purification Kit (Qiagen) and quantified in a Nano Drop Spectrophotometer 2000. The restriction-ligation reaction was performed as described above. The correct assemblies of the GBparts p*MtANR and pMtLAR* were confirmed by restriction analyses and sequencing.

### Transient expression assays in *N*. *benthamiana*

For transient plant transformation assays, plasmids were transferred to *A*. *tumefaciens* strain GV3101 by electroporation. Agroinfiltration was performed as previously described [41]. Briefly, cultures were inoculated in LB medium and incubated for 18-22h at 28°C and 250 rpm. Bacterial cultures were centrifuged for 20min at 4,000rpm and the pellets resuspended into agroinfiltration medium (10 mM MES pH 5.6, 10 mM MgCl_2_, 200 mM acetosyringone) to an optical density of 0.5 at 600 nm. Agroinfiltration was carried out using a needle-free syringe in leaves of 4–5 weeks old *N*. *benthamiana* plants. The vector pEGB 35S:DsRed:TNos [34], which product shows red fluorescence under UV light, was used as control to verify that agroinfiltration was functional. Two rounds of 10 plants were agroinfiltrated. Control plants were agroinfiltrated only with the reporter gene *DsRed*. Leaves were harvested 5 to 7 days post-infiltration and assayed for transgene expression.

### Stable genetic transformation of *N*. *tabacum*

The binary vector *pDGB2-Hyg-35S*:*AmRosea1*:*TNos-35S*:*AmDelila*:*TNos-35S*:*MtANR*:*TNos-35S*:*MtLAR*:*TNos* was electroporated into *A*. *tumefaciens* strain LBA4404. Tobacco plants were transformed according to standard procedures [42, 43] using leaf disks in co-culture with *A*. *tumefaciens* strain LBA4404. Transgenic tobacco plants were selected on organogenic induction medium IKZ containing 25 μg mL^-1^ hygromycin and 0.8% phytagar. Positive transgenic plants were confirmed by amplifying the *AmRosea1*, *AmDelila*, *MtANR* and *MtLAR* genes using genomic PCR. Specific primers for the *AmRosea1* (Rosea 244 Dir and Rosea 618 Rev), *AmDelila* (Delila Dir and Delila Rev), *MtANR* (MtANR 39 Dir and MtANR 419 Rev) and *MtLAR* (MtLAR 39 Dir and MtLAR 356 Rev) were used (S1 Table). Hygromycin resistant plants were acclimated and maintained in a greenhouse. Expression of the transgenes was confirmed in the positive transformants by qRT-PCR with RNA extracted from leaves. Plants with the complete set of transgenes in a single copy were retained for further analyses.

### RNA extraction

Immature seeds were collected from *M*. *truncatula* pods at developmental stage 10 days after anthesis using a stereomicroscope (Leica, MZ16F). Immature seeds were pulverized and resuspended in ice-cold RNA lysis solution of Plant RNA Purification Reagent (Invitrogen). Purified RNA was obtained as described in the Turbo DNA-free instructions (Ambion) after removing traces of contaminating DNA. The cDNA samples were synthesized from 1 μg of total RNA using the Primescript^™^ RT Reagent Kit (Perfect Real Time, Takara) and random primers in 20 μL total volume reaction. This cDNA was used to isolate the *MtANR* and *MtLAR* genes. RNA was also isolated from *N*. *benthamiana* and *N*. *tabacum* leaves using the same protocol described above. Total RNA was purified from tissue samples using the E.Z.N.A. Plant RNA Kit, following the manufacturer's recommendations (Omega) from 100 mg of tissue. RNA was quantified using absorbance at 260 nm, whereas its purity was assessed based on absorbance ratios at 260/280 nm. The integrity of purified RNA was confirmed by denaturing agarose gel electrophoresis and ethidium bromide staining. Then, 1 μg of total RNA was reverse transcribed with a Primescript™ RT Reagent Kit.

### RT-PCR analyses

All RT-PCR reactions were performed and analyzed on a Thermo Scientific Arktik Thermal Cycler (Thermo Fisher Scientific). The reaction mix was prepared in a 25 μL total volume containing 2 μl of cDNA solution, 0.2 mM of each dNTP, 3 mM MgCl_2_, 1x reaction buffer, 1 unit of Taq DNA Polymerase (Biotools) and 0.8 mM of the appropriate pair of primers (S1 Table). PCR conditions for amplification of *AmRosea1*, *AmDelila*, *MtANR*, *MtLAR*, *CHS*, *CHI*, *F3H*, *DFR1* and *ANS* consisted of initial denaturation at 94°C for 5 min, 30 amplification cycles of 94°C/30 sec, 55°C/30 sec, and 72°C/60 sec, and a 7 min final extension at 72°C. The PCR program was limited to 25 cycles for the housekeeping genes (*i*.*e*., *NtACT*; [44]). 20 μl of the PCR products were separated on 1% ethidium bromide-stained agarose gels. PCR fragments were visualized under UV using a Syngene GBOX (Syngene) and captured with the GeneSnap program (Syngene). The analyses were carried out in duplicate using biologically independent material with similar results. The possibility of genomic DNA contamination in the RT-PCR assays was controlled with the No Reverse Transcriptase control (No RT control). Oligonucleotides for the RT-PCRs were designed using the corresponding sequences of the *NtCHI*, *NtF3H*, and *NtANS* genes of *N*. *tabacum* and the *NbCHS* and *NbDFR1* genes of *N*. *benthamiana* (S1 Table).

### Extraction and quantification of soluble proanthocyanidins by the dimethylaminocinnamaldehyde (DMACA) reagent

The extraction of soluble proanthocyanidins from *N*. *benthamiana* leaves was performed as described by [45]. Briefly, 2 g of fresh material were ground in liquid nitrogen and extracted with 8 mL of extraction solution (70% acetone and 30% of 1% acetic acid in water). The extracts were vigorously vortexed for 2 min, sonicated at 30°C for 30 min, and then centrifuged at 8,000 rpm for 10 min to remove cell debris. The pellet was resuspended in 4 mL of extraction solution, repeating the same procedure. Supernatants of both extractions (12 mL) were collected and transferred to 25 mL glass flasks and acetone was evaporated under reduced pressure. The remaining aqueous phase was extracted twice with 10 ml of dichloromethane and three times with hexane. Samples were finally evaporated to dryness and dissolved in 1 mL of the first extraction solution for colorimetric reaction. Soluble PAs were quantified by reaction with the *p*-dimethylaminocinnamaldehyde (DMACA) reagent using catechin standards, as described previously [46, 47]. The DMACA reagent gives coloured adducts with flavanols showing maximum absorption between 632 and 640 nm, thus preventing the interference of other coloured compounds that might be present in the same extracts, such as anthocyanins. A 20 μL aliquot from the final extract with a concentration of 2 g of fresh *N*. *benthamina* leaves/mL, was mixed with 980 μL of DMACA reagent (0.2% [w/v] in a mixture of methanol and 3N HCl [1:1]) in spectrophotometer cuvettes. The reference blanks contained 20 μL of extraction solution instead of the aliquot samples. The absorbance at 640 nm was measured after 5 min using a Pharmacia Biotech Ultrospec 1000E UV/Visible spectrophotometer and PAs content was calculated, after subtracting the blanks, as catechin equivalents.

### Analysis of PAs by phloroglucinolysis and HPLC-MS

The phloroglucinolysis analysis allowed the quantification of proanthocyanidins after degradation in the presence of phloroglucinol that leads to the monomers (catechin and epicatechin as the terminal units of the oligomers) and the catechin and epicatechin adducts that show the extension units in the PAs. Proanthocyanidins were quantified as previously reported [48] using an acid catalysis in the presence of phloroglucinol. Briefly, 50 mg of lyophilized sample were dissolved in 800 mL of phloroglucinol (50 mg mL^-1^) added with ascorbic acid (10 mg mL^-1^) dissolved in methanol acidified with 0.1 N HCl. The reaction mix was vortexed and incubated at 50°C for 20 min. The reaction tube was placed in ice and 1 mL of 40 mM sodium acetate was added to stop the reaction. The sample was centrifuged, filtered with a 0.22 μm PVDF filter, and injected in an HPLC/MS apparatus. The identification and quantification of catechin, epicatechin and their adducts was carried out by Agilent 1100 Series apparatus equipped with detector MSD Trap 1100 Series (Agilent), as previously described [49, 50]. Briefly, the column used was an Atlantis C18 (250 mm x 4.6 mm, 5 μm particle size; Water, Milford, MA, US) operating at a flow rate of 1 mL min^-1^; the injection volume was 8 μL. The solvents were 2.5% acetic acid in water (A) and acetonitrile (B) with a separation gradient starting with 3% B in A at 0 min, 9% at 5 min, 16% at 15 min, 50% at 45 min followed by washing and conditioning steps. The phenolic compounds were quantified at 280 nm with a calibration curve of catechin (1–300 μg L^-1^). The MS detector operated in negative ion-mode. The Trap interface and ion optics settings were the following: spray potential 65 psi; nebulization gas (nitrogen) relative flow value 11; capillary temperature 325°C. Full-scan mass spectra were acquired scanning the range 100–800 m/z. For calculation of the degree of polymerization (mDP), the flavan-3-ol monomers (catechin and epicatechin) present endogenously in the plant material, were analyzed by the same chromatographic method used for the phloroglucinolysis analysis, before the phloroglucinolysis degradation and therefore the monomers present endogenously were substracted in the calculation of mDP.

## Results

### Generation of the GoldenBraid 2.0 multigenic construct

**Fig 1:**
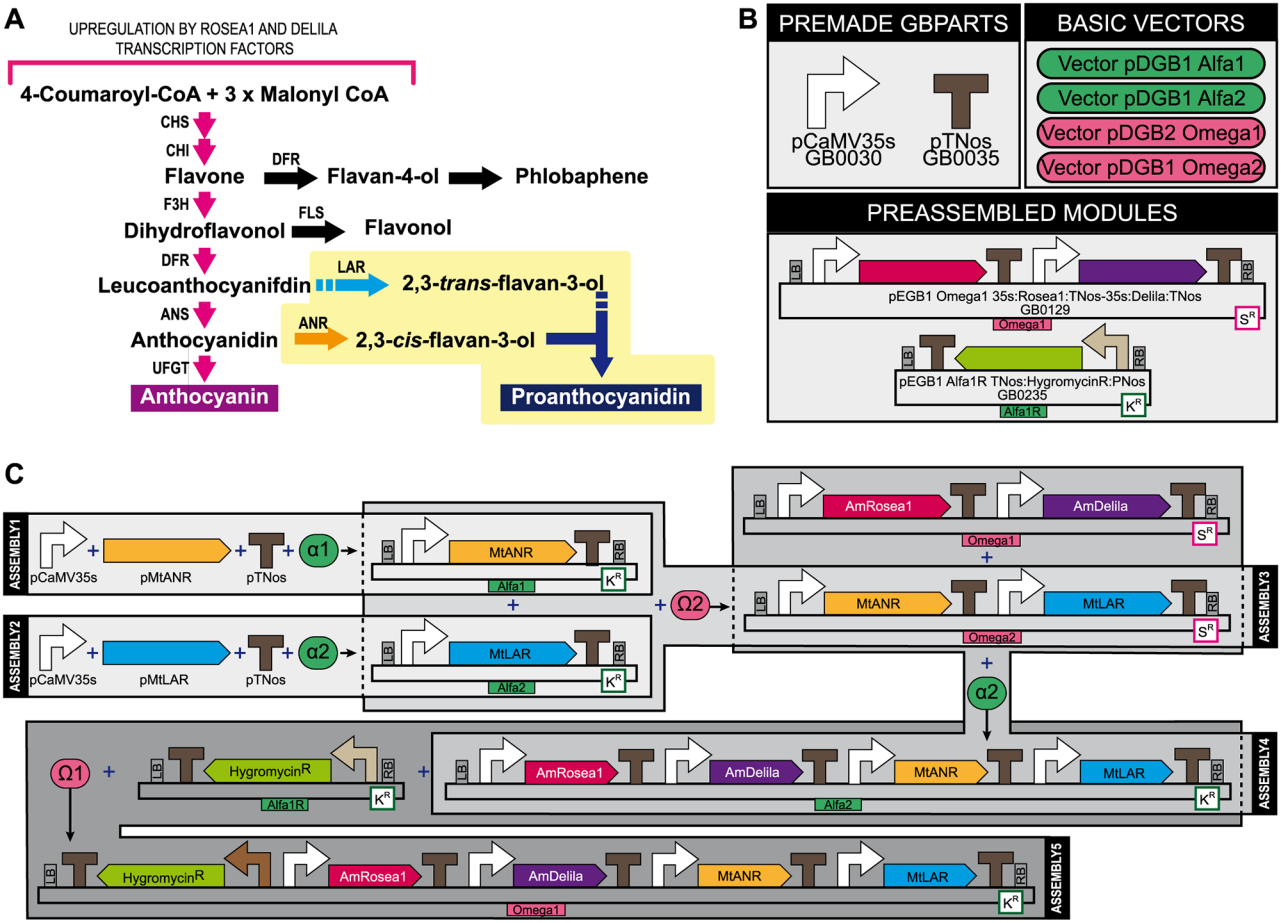
Anthocyanin and proanthocyanidin biosynthetic pathways and multigenic construct assembly strategy. **(A)** Schematic representation of the biosynthetic pathways for anthocyanins and proanthocyanidins. Abbreviations: chalcone synthase (CHS); chalcone isomerase (CHI); dihydroflavonol reductase (DFR); flavanone 3-hydroxylase (F3H); flavonol synthase (FLS); leucoanthocyanidin reductase (LAR); anthocyanidin synthase (ANS); anthocyanidin reductase (ANR); uridine diphosphate glucose-flavonoid 3-*O*-glucosyl transferase (UFGT). Purple colored arrows represent the catalytic steps that are upregulated by the overexpression of the *A*. *majus* transcription factors *Rosea1 and Delila*. The yellow highlighted area indicates the catalytic steps that are overexpressed in this work by the *M*. *truncatula* genes introduced in our multigenic construct, whose catalytic steps are highlighted in darker yellow and blue. **(B)** Premade GBParts, Modules and vectors used in this work. This includes the parts pCaMV35S promoter (GB0030), pTNos (GB0035), three vectors of the pGreenII-based pDGB1 series (Alfa1, Alfa2 and Omega2), one vector of the pCAMBIA pDGB2 series (Omega1) and two preassembled modules that were previously tested by the GB2.0 developers. The first module (GB0129) expresses the two *A*. *majus* transcriptional factors *Rosea1* and *Delila* that under the control of the CaMV35S promoter. The second module (GB0235) is the hygromycin resistant cassette that is used to select the transformed plants in the stable transformation process. CaMV35S is the Cauliflower Mosaic Virus 35S Promoter; TNos is the Nopaline synthase terminator; PNos is the Nopaline synthase promoter; K^R^ and S^R^ stand for bacterial kanamycin and spectinomycin resistance cassettes; LB and RB represent the Left and Right Borders of the T-DNA. **(C)** GoldenBraid 2.0 multigenic construct *AmRosea1*:*AmDelila*:*MtANR*:*MtLAR* generated in this work. The multigenic construct was generated in five steps that include the assembly of the *MtANR* and *MtLAR* transcriptional units from its basic parts (Assemblies 1 and 2), the combination of these transcriptional units in a single vector (Assembly 3), the later addition of the *A*. *majus* transcriptional factors to the *M*. *truncatula* genes (Assembly 4) and finally the incorporation of the hygromycin resistance cassette to generate the multigenic construct that is used in all the experiments of this work. *MtANR* is the *M*. *truncatula* anthocyanidin reductase gene; *MtLAR* is the *M*. *truncatula* leucoanthocyanidin reductase gene.

We have generated a multigenic construct using the GoldenBraid 2.0 modular cloning system to simultaneously activate the anthocyanin and proanthocyanidin pathways in plants. For this purpose we have combined the *AmRosea1* and *AmDelila* transcription factors of *A*. *majus* with the *anthocyanidin reductase* (*MtANR*) and the *leucoanthocyanidin reductase* (*MtLAR*) genes of *M*. *truncatula* to produce the two enzymes that function at branches between anthocyanin and PA biosynthesis (Fig 1A).

To generate the multigenic construct, first the *MtANR* and *MtLAR* genes were domesticated as GBparts, as described in the Materials and Methods section (Fig 1B). Next, two transcriptional units (TUs) were assembled from its basic parts (*i*.*e*., promoter+coding sequence+terminator) into the Alfa level plasmids (35S:MtANR:TNos into the pDGB1 Alfa1 and 35S:MtLAR:TNos into pDGB2 Alfa2, see Assemblies 1 and 2 in Fig 1C). The GBparts GB0030 (pCaMV35S, the Cauliflower Mosaic Virus 35S Promoter) and GB0035 (pTNos, the Nopaline synthase terminator) were used in the constructs with the p*MtANR* and p*MtLAR* genes. Both assembled TUs were further binary combined into the pDGB1 Omega2 vector (see Assembly 3 in Fig 1C), so that the generated construct is compatible with the preassembled 35S:AmRosea1:TNos-35S:AmDelila:TNos module, that was already available in the GBCollection (GB0129; https://gbcloning.upv.es/search/). The four genes were later combined into the pDGB1 Alfa2 vector (see Assembly 4 in Fig 1C). This four-gene construct was moved together with the hygromycin resistance gene (GB246) into the pDGB2 Omega1 Vector. This final multigenic construct was electroporated into *A*. *tumefaciens* strains GV3101 and LBA4404 and used for transient and stable transformation experiments.

### Functional validation of the multigenic construct by transient expression assays in *N*. *benthamiana*

**Fig 2:**
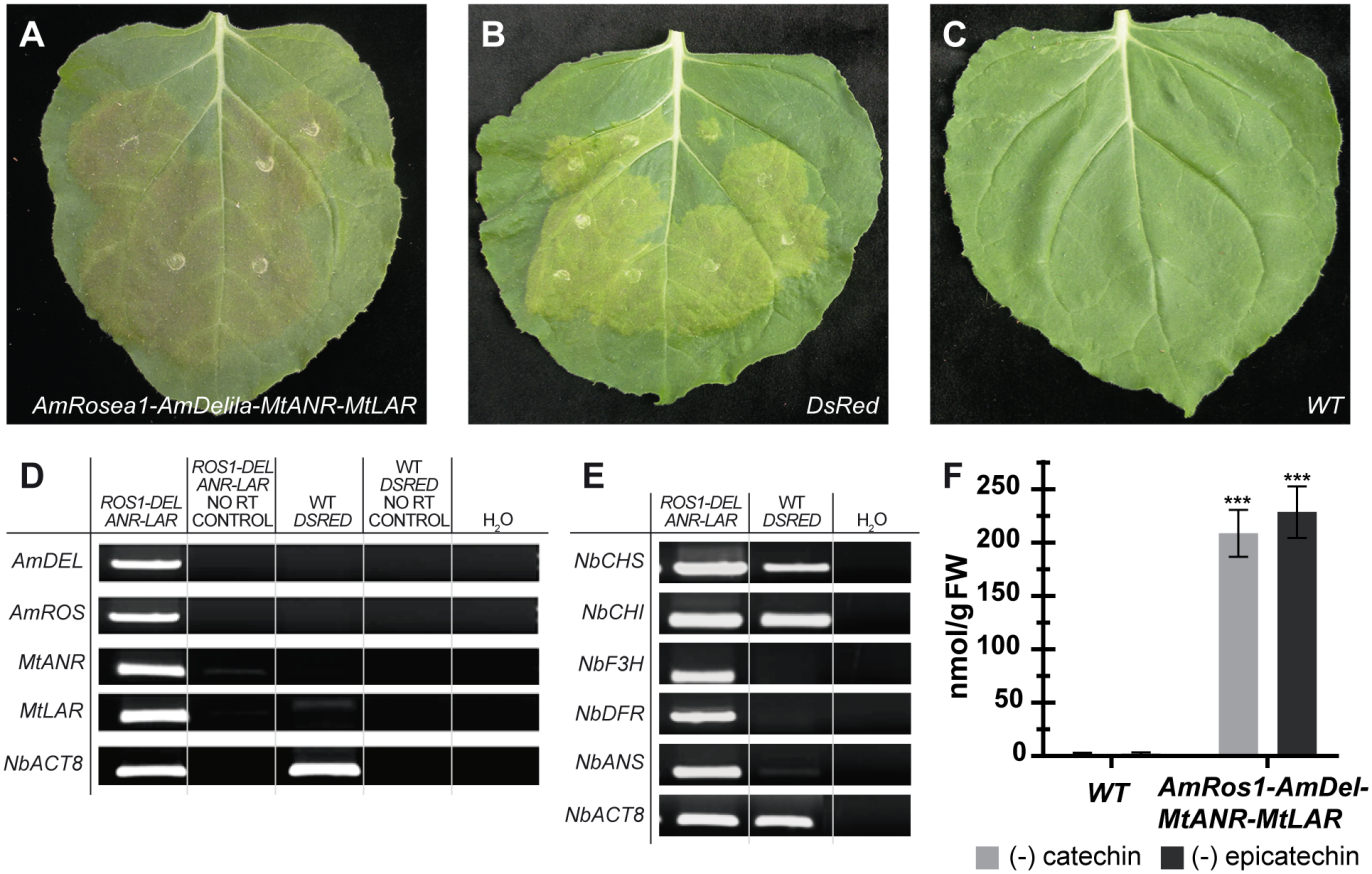
Validation of the multigenic construct by transient expression in *Nicotiana benthamiana* leaves. **(A)** Agroinfiltrated *N*. *benthamiana* leaf with the *AmRosea1*:*AmDelila*:*MtANR*:*MtLAR* multigenic construct showing purple pigmentation. **(B)** Infiltrated leaf with *DsRed* (control). **(C)** Non-infiltrated wild-type leaf. **(D)** Expression levels of the *AmRosea1*, *AmDelila*, *MtANR* and *MtLAR* transgenes in agroinfiltrated leaves of *N*. *benthamiana*. *NbACT8* is used as positive control. PCR results were obteined after 30 amplification cycles for all analized genes. **(E)** RT-PCR analysis of the anthocyanin biosynthetic pathway genes in agroinfiltrated leaves of *N*. *benthamiana*. The PCR results were obteined after 30 amplification cycles for all genes and 25 cycles for the housekeeping *NbACT8* gene. **(F)** PA levels in agroinfiltrated leaf extracts of *N*. *benthamiana* obteined by the dimethylaminocinnamaldehyde (DMACA) colorimetric reaction. The DMACA reaction showed an increase of the PA’s content in the agroinfiltrated leaf area compared with the non-infiltrated WT leaves. The maximum PA levels were found in the plants infiltrated with the multigenic construct. Statistical T-Test values for (-) catechin (n = 3, t = 11.46, df = 4, *p* = 0.0003) and (-) epicatechin (n = 3, t = 11.46, df = 4, *p* = 0.0003).

To test the functionality of the complete multigenic construct *AmRosea1*:*AmDelila*:*MtANR*:*MtLAR*, we performed transient expression assays in agroinfiltrated leaves of *N*. *benthamiana* plants. Our results showed purple pigmentation (anthocyanin production) in the infiltrated areas (Fig 2A) in comparison with the infiltrated control with the *DsRed* reporter gene and the non-infiltrated WT control (Fig 2B and 2C respectively). The purple pigmentation was evident 5 days after infiltration and increased progressively.

To determine the expression levels of *AmRosea1*, *AmDelila*, *MtANR* and *MtLAR*, we performed RT-PCR analyses using RNA isolated from the *N*. *benthamiana* agroinfiltrated leaves. The housekeeping gene *Actin-8* (*NbACT8*) was used as control of a constitutive expression. Our results indicated that the CaMV35S constitutive promoter induced high expression of the four transgenes after 25 amplification cycles in leaves collected 5 days after infiltration (Fig 2D). In addition, a RT-PCR assay demonstrates that the increased expression of both *AmRosea1* and *AmDelila* transcription factors is able to upregulate the expression of the genes involved in the anthocyanin biosynthetic pathway (*CHS*, *CHI*, *F3H*, *DFR* and *ANS*). Our results show that an increase in the expression levels both TFs specially activates the *F3H*, *DFR1* and *ANS* genes when compared with control leaves infiltrated with *DsRed*, in which these genes were not activated (Fig 2E). This analysis corroborated the regulatory effect of both *AmRosea1* and *AmDelila* TFs in the expression of three central enzymes of the flavonoid biosynthetic pathway: CHS, F3H and ANS [17].

### Detection of flavan-3-ols and proanthocyanidins in agroinfiltrated *N*. *benthamiana* leaves

DMACA assays showed increased PA levels in the *N*. *benthamina* plants infiltrated with the *AmRosea1*:*AmDelila*:*MtANR*:*MtLAR* multigenic construct compared with the non-infiltrated WT control. Total flavonoid content was expressed as catechin or epicatechin equivalents [228.51 ± 24.17 nmol (-)-epicatechin/g FW and 208.56 ± 31.19 nmol (-)-catechin/g FW respectively], (Fig 2F and S2 Table).

### Experimental validation of the multigenic construct by stable transformation of *N*. *tabacum*: Integration and expression of transgenes

The validated multigenic construct *AmRosea1*:*AmDelila*:*MtANR*:*MtLAR* was used to perform stable genetic transformation experiments in *N*. *tabacum*. Two important aspects were evaluated in the regenerated T0 transgenic plants: the integration capacity of the four transgenes (which is especially important for multigenic constructs) and their respective expression levels.

**Fig 3:**
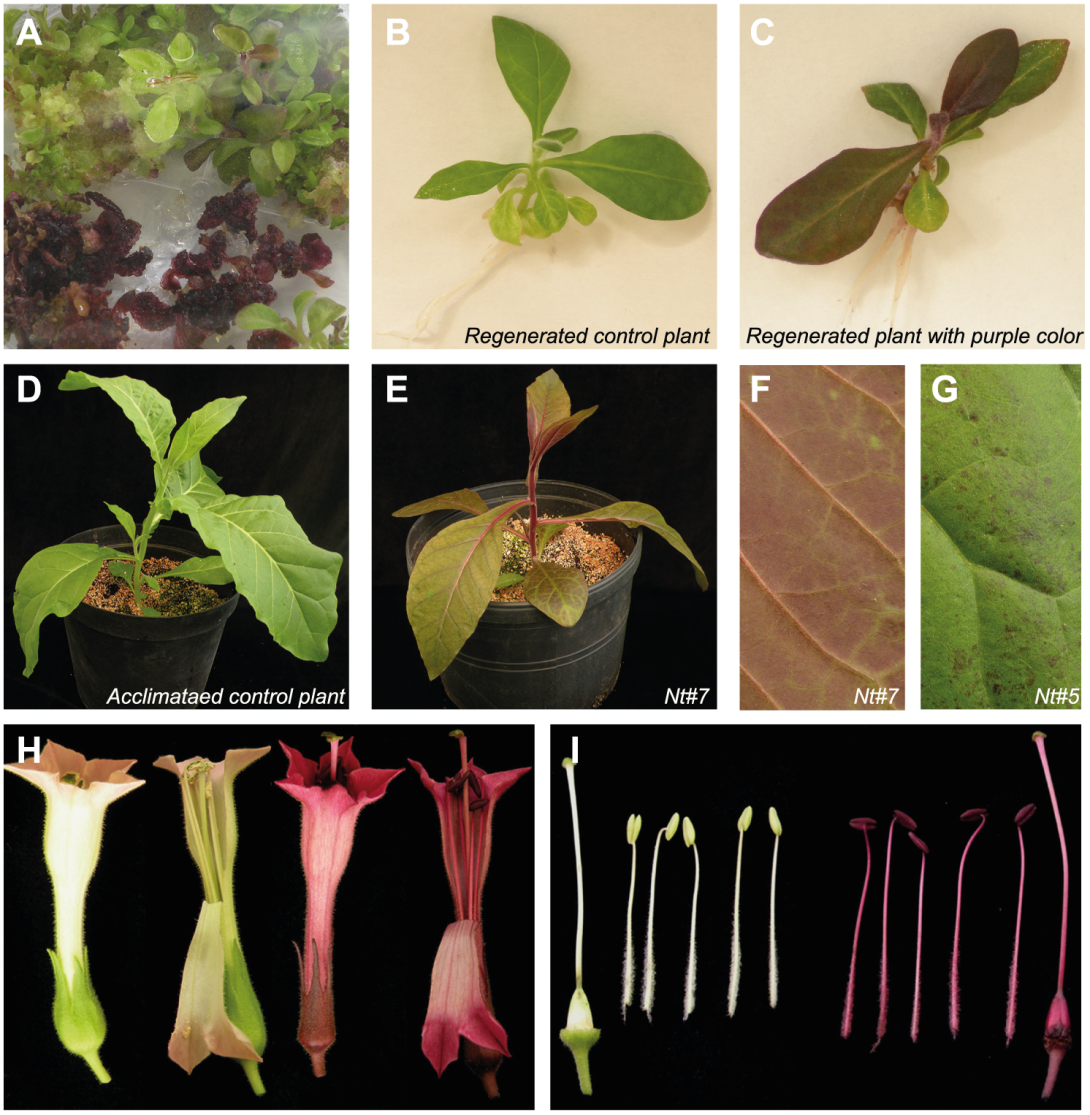
Phenotypes of the *AmRosea1*:*AmDelila*:*MtANR*:*MtLAR* transgenic tobacco plants. **(A)** Some of the *in vitro* regenerated transgenic calli showed an intense purple pigmentation due to accumulation of anthocyanins. **(B)** *In vitro* regenerated control plantlet. **(C)** *In vitro* regenerated transgenic tobacco plant showing intense purple color in all tissues. **(D)** Control plant after acclimation in the greenhouse. **(E)** Transgenic tobacco plant Nt#7 with intense purple pigmentation after acclimation in the greenhouse. **(F)** Detail of a leaf from the transgenic plant Nt#7 showing intense purple pigmentation in the abaxial side and vascular bundles. **(G)** Detail of a leaf from the trasngenic plant Nt#5 showing only small patches of purple pigmentation. **(H)** Entire and disected flower from a control (left) and transgenic plant Nt#7 (right). **(I)** Carpel and stamens from a disected flower of a control (left) and the Nt#7 transgenic plant (right).

Anthocyanin pigmentation was a visual sign for the selection of hygromycin resistant tobacco plants. We detected high levels of anthocyanin pigmentation in the young developing *N*. *tabacum* transgenic calli, however some of them never regenerated shoots (Fig 3A). This effect is probably due to the toxic effects of the excess in anthocyanin production during the regeneration process [51, 52]. In the pool of regenerated plants, we selected 10 plants from different transgenic lines. Most of them showed more or less severe purple pigmentation in all plant tissues (stem, leaves, flowers) when compared with control plants (Fig 3B–3F, 3H and 3I). One plant (Nt#5) showed small purple spots in the leaves (Fig 3G, in comparison with a fully purple leaf from plant Nt#7 in panel F). We observed that plant growth was affected in those plants where higher purple pigmentation occurred. These findings suggest that high levels of anthocyanin accumulation, mainly produced by the ectopic expression driven by the 35S promoter, have a deleterious effect on plant growth and development.

The integration capacity of the multigenic construct into the plant genome was proved by PCR amplification of genomic DNA isolated from young leaves of *N*. *tabacum* hygromycin-resistant plants using specific oligonucleotides (S1 Table). Our results showed the presence of the four transgenes in six (Nt#5, #6, #7, #8, #9 and #10) of the 10 *N*. *tabacum* transgenic plants analyzed (S1A Fig). These results suggest that the integration capacity of the complete set of transgenes into the *N*. *tabacum* genome is high.

### Transgene expression levels in T0 *N*. *tabacum* plants

**Fig 4:**
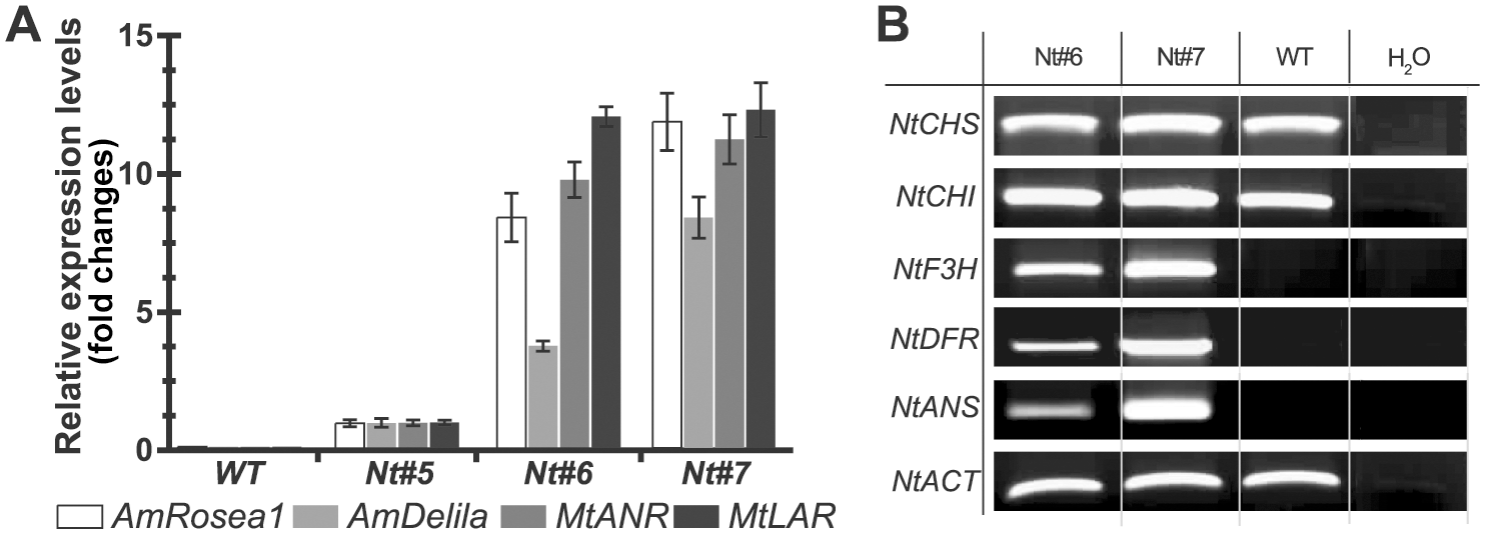
Expression analyses of transgenes and key genes involved in the anthocyanin biosynthetic pathway in transgenic *Nicotiana tabacum* leaves. **(A)** qRT–PCR analysis of *AmRosea1*, *AmDelila*, *MtANR* and *MtLAR* transgenes in transformed leaves of *N*. *tabacum*. Error bars correspond to the standard deviation of three replicates. The expression value of *AmRosea1* in plant Nt#5 was set to 1.00 and the expression levels of the rest of transgenes were plotted relative to this value. To normalize the samples the constitutive *NtACT8* gene was used. **(B)** RT-PCR expression analysis of key genes involved in the anthocyanin pathway in *N*. *tabacum* Nt#6 and Nt#7 transgenic plants. PCR results were obtained after 30 amplification cycles for all genes and 25 cycles for the housekeeping *NtACT8* gene.

The expression levels of the four transgenes were evaluated by quantitative RT-PCR (qRT-PCR) in young leaves of *N*. *tabacum*, establishing as constitutive expression the endogenous *Actin-8* gene (*NtACT*). Three *N*. *tabacum* plants were selected according to their phenotype: one plant presenting only small purple spots in their leaves (Nt#5), one plant showing a partial purple pigmentation (Nt#6) and one plant showing strong purple pigmentation in all tissues (Nt#7). Our results showed that the expression level of the four transgenes varies between the different transgenic lines (greater in Nt#7 and lower in Nt#5, Fig 4A). This finding also correlated with the different intensity in anthocyanin accumulation, which depends on the expression levels of the *AmRosea1* and *AmDelila* transcription factors.

We also analyzed the expression levels of these genes encoding the main enzymes required for anthocyanin biosynthesis in the T0 *N*. *tabacum* plants, which should have been upregulated by the expression of *AmRosea1* and *AmDelila*. RT-PCRs were performed using RNA from leaves of transgenic *N*. *tabacum* plants Nt#6 and Nt#7. The endogenous *NtACT* gene was used as control of constitutive expression. In *N*. *tabacum*, there was a correlation between the expression levels of the transgenes (Fig 4B) and the severity of the coloured phenotypes observed. In addition, our results showed that *AmRosea1* and *AmDelila* upregulated the expression of genes encoding the enzymes acting in the central route of flavonoids biosynthesis (*NtF3'H*, *NtDFR1* and *NtANS*) in the *N*. *tabacum* transgenic plants, when compared with control plants.

### Detection of flavan-3-ols and proanthocyanidins in transgenic *N*. *tabacum* leaves

**Fig 5:**
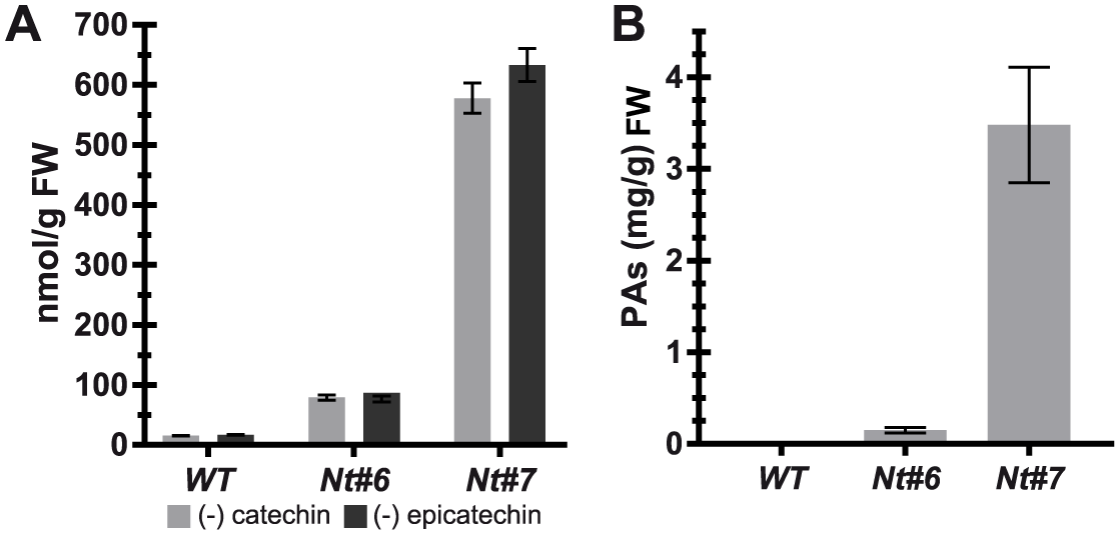
Proanthocyanidin levels in *Nicotiana tabacum* transgenic plants. **(A)** Determination of proanthocyanidin levels in leaf extracts of *N*. *tabacum* Nt#6 and Nt#7 transgenic plants by dimethylaminocinnamaldehyde (DMACA) assays. Results show a signigicant increase of PA content in the transgenic leaves when compared with the wild-type plant. **(B)** Determination of PA levels in leaf extracts of transgenic *N*. *tabacum* Nt#6 and Nt#7 plants by phloroglucinolysis. Results show a great increase of total PA levels in the transgenic leaves of plant Nt#7 when compared with plant Nt#6 and a wild-type plant. Data represent the average of three replicates and the standard error for each sample.

We analyzed whether the constitutive expression of the four transgenes *AmRosea1*, *AmDelila*, *MtANR* and *MtLAR* were able to activate the route of biosynthesis of PAs in two *N*. *tabacum* transgenic plants that showed partial and severe purple pigmentation (Nt#6 and Nt#7 respectively). Colorimetric analysis of leaf extracts with DMACA showed that both plants produced more PAs when compared with a WT control plant. The maximum levels of PAs expressed as catechin or epicatechin equivalents were found in the plant Nt#7 (633 ± 27.5 nmol (-) epicatechin/g FW and 577.79 ± 25.09 nmol (-) catechin/g FW), (Fig 5A and S3 Table). Plant Nt#6 showed seven-fold less production of PAs than plant Nt#7 (86.56 ± 4.91 nmol (-) epicatechin/g FW and 79 ± 4.48 nmol (-) catechin/g FW). These results corroborate the active role of the enzymes MtLAR and MtANR in the transgenic plants catalyzing the conversion of their respective intermediate substrates (leucoanthocyanidin and anthocyanidin) in proanthocyanidins.

The PAs content was determined by phloroglucinol derivatization (phloroglucinolysis). Phloroglucinolysis allowed the quantification of PAs after separation of the different products (terminal units and adducts) in the presence of phloroglucinol, followed of HPLC-MS analysis. This analysis showed that only epicatechin was present as extension unit, and also that epicatechin is more relevant than catechin. As expected, in plants Nt#6 and 7 we detected the presence of PAs when compared with a WT control plant. The high level of PAs was detected in plant Nt#7 (3.48 mg/g DW), in contrast with plant Nt#6 (0.15 mg/g DW) (Fig 5B). We also detected differences in the average degree of polymerization (mDP) in plants Nt#6 and Nt#7. Plant Nt#6 presents the highest value (2.32 ± 0.12), compared with plant Nt#7 (1.76 ± 0.56). This result indicates that PAs in Nt#7 are mainly composed of monomers and dimers, whereas in Nt#6 is mainly composed of dimers but in small quantities (S4 Table).

All the exposed results revealed that there is a correlation between the expression levels of *AmRosea1* and *AmDelila*, the activation of genes encoding the enzymes involved in the anthocyanin biosynthetic pathway, the purple pigmentation of the different plant tissues and the *de novo* production of PAs in the *N*. *tabacum* transgenic plants. All together indicate that the multgenic construct is fully functional and simultaneously activate the anthocyanin and proanthocyanidin biosynthetic pathways.

### Heritability and functionality of the four transgenes in the T1 tobacco plants

**Fig 6:**
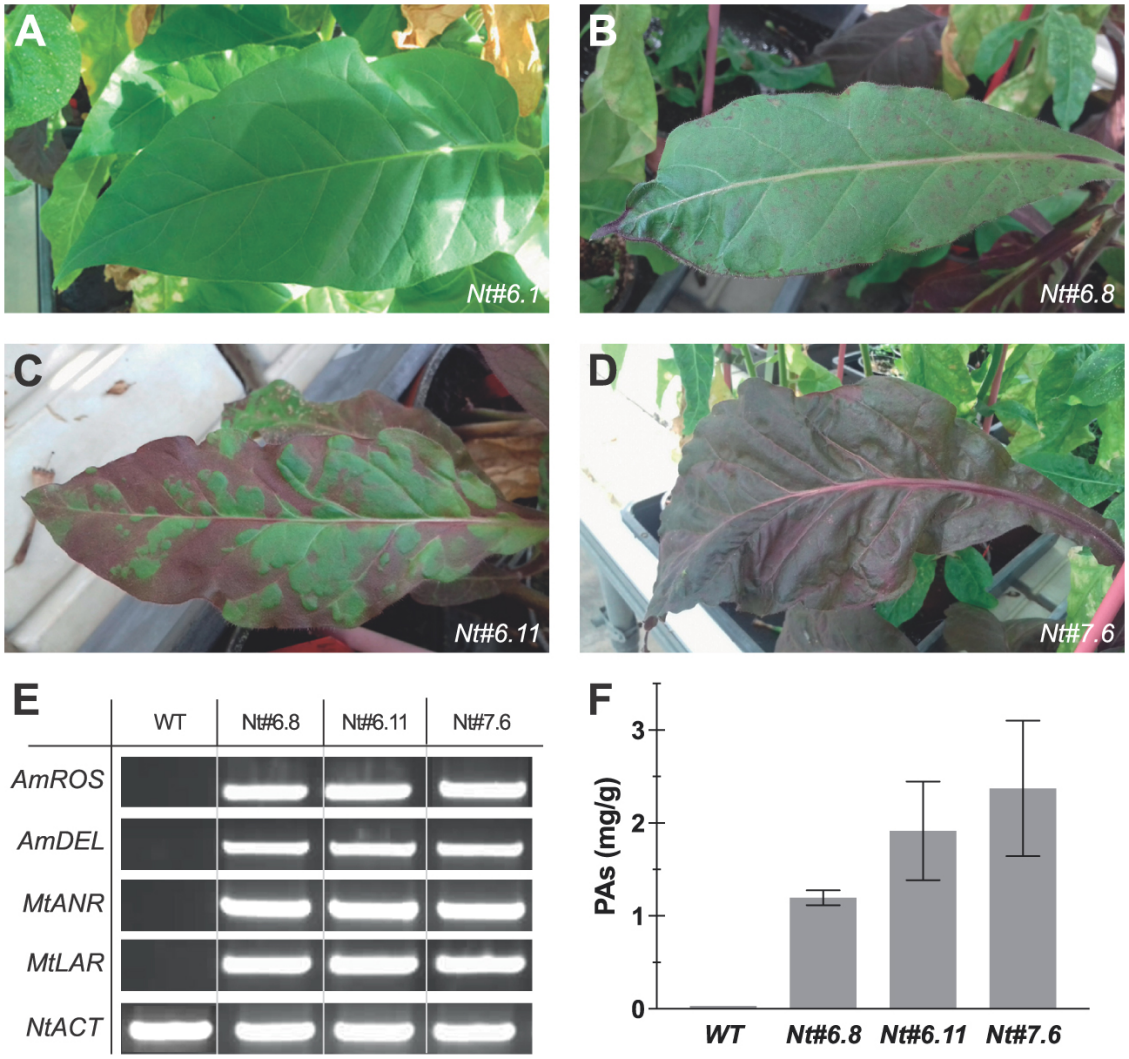
Relationship between purple phenotype, transgene expression and PAs production in three T1 *Nicotiana tabacum* plants. **A-D.** Different leaf coloured phenotypes in the T1 of *N*. *tabacum* transgenic plants Nt#6.1 (green), Nt#6.8 (purple spots), Nt#6.11 (purple patches) and Nt#7.6 (full purple). **E.** In the lineage of plants Nt#6 and Nt#7 we analyzed by semi-qRT-PCR three plants with the complete set of transgenes showing a weak (Nt#6.8), a middle (Nt#6.11) and a strong purple phenotype (Nt#7.6). In the three plants the four transgenes were properly expressed. To normalize the samples the constituve *NtACT8* gene was used. **F.** The Nt#6.11, Nt#6.8 and Nt#7.6 transgenic plants produced PAs as demonstrated by HPLC-MS analysis of leaf extracts when compared with the WT.

All the tobacco T0 plants selected were fertile and produced seeds. The heritability of the four transgenes was evaluated in the second generation (T1) of *N*. *tabacum* Nt#6 and Nt#7 transgenic plants (S1B Fig). In both cases the lineages obtained showed different degrees of coloured leaf phenotypes (Fig 6A–6D). The purple plants showing a strong phenotype (high anthocyanin content) grew more slowly than those showing middle or weak purple phenotypes probably due to the excessive accumulation of anthocyanins in their vacuoles. About the 60% of the T1 plants incorporated the full set of transgenes. In the lineage of plant Nt#6, the unique expression of *AmRos1* in the Nt#6.2 plant was sufficient to induce anthocyanin production, whereas plants Nt#6.1 and Nt#7.5 showed a green phenotype due to the absence or low expression of *AmRos1*. The unique presence of *AmDel* was not capable to induce anthocyanin production in the Nt#6.1 plant. The purple plants showing a strong phenotype grew more slowly during the first stages of development than those showing middle or weak purple phenotypes, probably due to the excessive accumulation of anthocyanins in their vacuoles as we indicated previously. In the lineage of plants Nt#6 and Nt#7 we analyzed by semi-qRT-PCR three plants with the complete set of transgenes showing a weak (Nt#6.8), a middle (Nt#6.11) and a strong purple phenotype (Nt#7.6) (Fig 6B–6D). In the three plants the four transgenes were properly expressed (Fig 6E). To normalize the samples the constituve *NtACT* gene was used. The Nt#6.8, Nt#6.11 and Nt#7.6 transgenic plants produced PAs as demonstrated by HPLC-MS analysis of leaf extracts when compared with the WT (Fig 6F and S5 Table). The high level of total PAs was detected in plant Nt#7.6 (2.37 mg/g DW), followed by plant Nt#6.11 (1.92 mg/g DW) and plant Nt#6.8 (1.20 mg/g DW). We also detected differences in the average degree of polymerization (mDP), plant Nt#7.6 presents the highest value (2.82 ± 0.14), followed by plant Nt#6.11 (2.37 ± 0.26) and by plant Nt#6.8 (2.22 ± 0.01).

## Discussion

Genetic modification of secondary metabolic pathways to produce desirable natural products is an attractive approach in plant biotechnology. The ability to manipulate the biosynthesis of flavonoids in plants is of high interest in the areas of nutraceuticals and human and animal feed. Proanthocyanidins are active principles of plants with demonstrated antioxidant properties to provide benefits to the health and contribute as anti-aging resources [53, 54].

Different MYB, bHLH and WD-40 complexes regulate the expression of genes encoding enzymes catalysing each step of the anthocyanin and PA biosynthetic pathways [55–57]. Butelli *et al*. [17] set out to produce tomato fruits with substantially elevated levels of anthocyanins by harnessing of two selected *A*. *majus* transcription factors (*AmRosea1* and *AmDelila*) under the control of the E8 fruit-specific promoter. The use of both TFs to induce anthocyanidin production and the co-expression of *leucoanthocyanidin reductase* (*MtLAR*) and *anthocyanidin reductase* (*MtANR*) from *M*. *truncatula*, might then lead to PA biosynthesis in plants. The DNA assembly platform GoldenBraid2.0, developed for multigene engineering, could be a good instrument to incorporate more than two genes in the same plasmid and therefore to stack genes in plants. Currently, there are no reports on the use of multigenic constructs to introduce a set of genes to activate the anthocyanin and PA pathways in the same transgenic plant.

### The GB2.0 multigenic construct transitory activates the anthocyanin and PA biosynthetic pathways in *N*. *benthamiana*

Using the *AmRosea1* and *AmDelila* TFs, in combination with two *M*. *truncatula* (*MtLAR* and *MtANR*) genes, we have generated a multigenic GoldenBraid2.0 construct to simultaneously activate both the anthocyanin and proanthocyanidin biosynthetic pathways. Transient expression experiments in *N*. *benthamiana* showed the activation of the anthocyanin pathway producing a purple color into the infiltrated leaves. Our results indicated that the increased expression of both *AmDelila* and *AmRosea1* TFs, is able to upregulate the expression of genes involved in the anthocyanin biosynthetic pathway *CHS*, *CHI*, *F3H*, *DFR1* and *ANS*, activating the *F3H*, *DFR1* and *ANS* genes coding for three central enzymes of the flavonoid biosynthetic pathway [58]. In addition, DMACA assays showed the *de novo* production of PAs in the *N*. *benthamina* plants infiltrated with the multigenic construct.

### The multigenic construct *AmRosea1*:*AmDelila*:*MtANR*:*MtLAR* simultaneously activates the anthocyanin and PA biosynthetic pathways in stably transformed *N*. *tabacum* plants

*N*. *tabacum* plants were stably transformed with the above described multigenic construct. One of the interesting aspects evaluated in the transgenic plants was the integration capacity of the four transgenes present in the construct. Full insertion of the four transgenes occurred in 60% of the tobacco plants. This information is relevant to the GoldenBraid 2.0 cloning system community of users, since few of the publications that have reported stably transformed plants analyze in depth the integrity of the multigenic construct through generations [37, 38, 40]. Our results suggest that there is a relationship between the integration capacity of the complete set of transgenes present in the multigenic construct and the plant species in question. The process of integration of the T-DNA often results in deletion, inversion or duplication of the DNA portion between the right and the left borders [59, 60]. Studies which demonstrate the relationship between the species and the loss of transgenes after the insertion of the T-DNA have been conducted in the last years. For example, the *nptII* gene showed different levels of deletion, being 12% in tobacco, 30% in watermelon and 60% in carrot [61]. In our multigenic construct the transgenes closest to the borders of the T-DNA are the gene that confers resistance to hygromycin (*hpt*) (left border, LB) and the *MtLAR* gene (right border, RB). In T0 *N*. *tabacum* plants, we observed always the deletion of *MtLAR* when located besides the RB. However, T1 *AmRosea1*:*AmDelila;MtANR*:*MtLAR* transgenic plants tend to lose neighbour transgenes located near the LB (*AmRosea1* or *AmDelila*). We never documented the loss of alternate transgenes. This deleterious effect could be connected to the repetitive use of the same promoter and terminator in all the transcriptional units (*i*.*e*., CaMV35S promoter and TNos terminator) so favouring DNA recombination that results in the loss of portions of the multigenic construct. The use of different promoters and terminators for multigenic constructs is highly suggested, and this could be easily implemented by browsing the GBCollection and using the different characterized regulatory regions [35, 40].

Regardless the percentage of plants that carry out the four transgenes and the deletion patterns of these in tobacco, the use of multigenic constructs generated by the GoldenBraid 2.0 cloning system is a suitable option for the stable transformation of plants. In this way, it is possible therefore the co-expression of multiple transgenes, indicating that this system is appropriate to modify polygenic characters in different plant species.

We evaluated whether the TFs *AmRosea1* and *AmDelila* were able to activate the route of biosynthesis of flavonoids in *N*. *tabacum*, with the consequent production of anthocyanins. Most of the transgenic plants presented purple pigmentation in all vegetative and reproductive tissues, with a greater accumulation into the abaxial side of the leaves and vascular tissues. Tobacco plants with an intense purple pigmentation in all tissues due to accumulation of anthocyanins, showed a delayed development. This fact can be explained because cells transport and store the produced anthocyanins in the vacuoles, which have a limited storage capacity. The accumulation of anthocyanins into the cells resulted in toxicity. The consequence is a delay in plant development and even plant death [51, 52]. After these results, we decided to investigate if the genes encoding the enzymes involved in the biosynthesis of flavonoids pathway were induced in the transgenic plants. Our results showed that the expression of genes encoding the enzymes CHS, CHI, F3´H, DFR1 and ANS, were induced in the transgenic plants indicating a correlation exists between the expression levels of the TFs *AmRosea1* and *AmDelila*, the purple phenotype and the expression of genes involved in the anthocyanin biosynthetic pathway. Thus, the TFs of Antirrhinum, *AmRosea1* and *AmDelila*, are able to recognize *cis* regulatory elements present in the promoters of *N*. *benthamiana* and *N*. *tabacum* genes encoding enzymes involved in the biosynthesis pathway of anthocyanins in both species.

The biosynthesis of PAs shares common steps with the anthocyanin biosynthetic pathway until the leucocyanidin step. Leucocyanidin is converted into flavan-3-ols catechin and epicatechin through either via a single-step reaction catalyzed by LAR or a two-step reaction catalyzed by leucoanthocyanidin dioxygenase (LDOX) and ANR, respectively [4]. Genetic engineering of catechin-based PAs requires operation of the anthocyanin pathway only as far as leucoanthocyanidin, whereas epicatechin-derived PAs require a source of anthocyanidin in addition to expression of *ANR* [19]. When Xie *et al*. [14] co-expressed the *MtANR* gene and the MYB transcription factor *AtPAP1* in tobacco, no free (+)-catechin was detected in the *AtPAP1-MtNANR* transgenic plants. Ectopic expression of the tea (*Camellia sinensis*) genes *CsANR2* or *CsLAR* led to the accumulation of low levels of PA precursors and their conjugates in *Medicago truncatula* hairy roots and anthocyanin-overproducing tobacco. Surprisingly, the expression of *CsLAR* in tobacco overproducing anthocyanin led to the accumulation of higher levels of epicatechin and its glucoside than of catechin, again highlighting the potential importance of epimerization in flavan-3-ol biosynthesis [21].

We used the colorimetric DMACA reaction to evaluate the presence of flavanols and PAs in leaf extracts from the *AmRos1*:*AmDel*:*MtANR;MtLAR* transgenic tobacco plants. In addition, the analysis by HPLC coupled to a mass spectrometer is the ideal method to analyze their content in PAs after phloroglucinolysis. Our results showed that the production of PAs (catechin and epicatechin) varied between the analyzed transgenic plants, being higher in the plant presenting more purple pigmentation which is associated with a greater accumulation of anthocyanins. It is also likely that the presence of PAs in *N*. *tabacum* is due to the enzymatic action of *MtANR* and *MtLAR*, being able to convert their relevant intermediate substrates in PAs. Leucoanthocyanidin reductase has been shown to convert leucocyanidin to (+)-catechin. *M*. *truncatula* has a single *LAR* gene that is highly expressed in the seed coat, but the PAs present in the seed coat are composed almost exclusively of epicatechin [27]. It has been reported that *MtLAR* has a role in the extension of proanthocyanidins, and a loss of function of this gene unexpectedly leads to loss of soluble epicatechin-derived PAs, increased levels of insoluble PAs, and accumulation of 4β-(S-cysteinyl)-epicatechin [62]. In the stable transformed tobacco plants with our multigenic construct, the catechin levels measured by phoroglucinolysis/HPLC-MS were lower when compared with the epicathechin ones. Likewise, the expression of other *LAR*s in transgenic plants also failed to result in the accumulation of cathechin or produced more epicathechin than cathechin [21, 27, 63–65].

Our results indicate that there is a correlation between the expression levels of *AmRosea1* and *AmDelila* and the activation of genes encoding the enzymes involved in the biosynthetic pathway of flavonoids, anthocyanin accumulation and production of PAs. In addition, we have corroborated the expression of the four transgenes in the T1 plants and the existence of a correlation in the anthocyanin accumulation and PAs production in the transgenic plants. All the exposed results lead to validate our multigenic construct to simultaneously activate the anthocyanin and proanthocyanidin pathways in two *Nicotiana spp*.

### Possible biotechnological applications of the multigenic construct: Activation of anthocyanin and PA biosynthetic pathways in forage legumes

Transcriptional regulation of flavonoid biosynthesis is poorly understood in legumes. Major forage legumes like clovers, alfalfa and lupine do not contain appreciable amounts of PAs in the leaves, where they only accumulate in glandular trichomes [66]. This is insufficient to prevent “pasture bloat” in ruminant animals, which is caused by the production of methane gas in the rumen due to excessive fermentation of dietary protein from forages. Modest levels of PAs in forages reduce the occurrence of bloat and at the same time promote increased dietary protein nitrogen utilization in ruminant animals [2, 66, 67]. The alfalfa (*M*. *sativa*) has high protein content but lacks PAs in their vegetative organs. The presence of PAs into alfalfa could eliminate help to fight pasture bloat, improve the efficiency of conversion of plant protein into animal protein (ruminal protein bypass), reduce greenhouse gases, reduce gastrointestinal parasites and inhibit insect feeding [68–74].

Classical breeding approaches have failed to introduce PAs into alfalfa foliage, and this problem is likely to require a biotechnological solution. Different genetic and metabolic engineering approaches to induce PAs production in forages have been reported in the last decade but have not been fully successful [21]. The majority of studies so far reported indicate that the regulation of PAs is more complex compared with anthocyanin biosynthesis and that provision of sufficient substrate and high expression of the *ANR* and *LAR* genes are still insufficient to support high levels of PAs accumulation in transgenic alfalfa plants [21, 58, 63, 75–78].

The concentration and structure of the PAs strongly affect the palatability (bitter taste) and nutritional value of forage legumes being in the order of 20–45 g/kg or 2–4.5% of dry weight the suitable quantities [75]. It has been suggested that a PAs concentration of about 20 mg/g DW in the forage might be sufficient to prevent frothy bloat in cattle [66, 68]. In the *N*. *tabacum* transgenic plants produced in this work the PAs concentration was lower with respect to the recommended concentration in forages. In any way, the production of PAs using our multigenic construct should be probed in transgenic alfalfa plants where the Medicago genes *MtANR* and *MtLAR* could be more efficient than in *Nicotiana spp*. due to the high degree of synteny existing between both legumes.

We have proved that the *AmRosea1* and *AmDelila* transcription factors of *A*. *majus* are completely functional in two *Nicotiana spp*. and, in combination with the two Medicago genes (*MtANR* and *MtLAR*), are able to induce PAs production in agroinfiltrated leaves and in stably transformed plants. This multigenic approach could be useful to generate transgenic alfalfa plants producing PAs. The versatility of the GB2.0 cloning system allows to easily incorporate new combinations of different transcription factors with other *ANR* and *LAR* genes to the multigenic scheme that could help to increase PAs production in the transgenic plants.

## Conclusions

1. We generated a GoldenBraid 2.0 multigenic construct containing two *A*. *majus* transcription factors (*AmRosea1* and *AmDelila*) to upregulate the anthocyanin pathway in combination with two *M*. *truncatula* genes (*MtLAR* and *MtANR*) to produce the enzymes that will derivate the biosynthetic pathway to PAs production.
2. Transient expression experiments by agroinfiltration of *N*. *benthamiana* leaves showed the activation of the anthocyanin pathway, producing a purple color in the infiltrated areas, the activation of genes involved in the anthocyanin biosynthetic pathway, and the effective production of PAs in comparison with non-infiltrated control plants.
3. The integration capacity of the four transgenes, their respective expression levels and their heritability were verified in T0 and T1 stably transformed *N*. *tabacum* plants.
4. DMACA and phoroglucinolysis/HPLC-MS analyses corroborated the activation of both pathways and the effective production of PAs in T0 and T1 transgenic tobacco plants in comparison with non-transformed control plants.

## Supporting information

S1 Fig Transgene integration and heritability in T0 and T1 *Nicotiana tabacum* transgenic plants. **(A)** Detection by PCR of the presence of the *AmRosea1*, *AmDelila*, *MtANR* and *MtLAR* transgenes in T0 *N*. *tabacum* transgenic plants. 6 out of 10 plants incorporated the complete full set of transgenes. Plants Nt#1 to Nt#4 lacked the *MtLAR* transgen. (**B)** Detection by PCR of the presence of the *AmRosea1*, *AmDelila*, *MtANR* and *MtLAR* transgenes in some plants of the T1 lineage of *N*. *tabacum* Nt#6 and Nt#7 transgenic plants. About the 60% of the T1 plants incorporated the full set of transgenes. The unique expression of *AmRosea1* in the Nt#6.2 plant was sufficient to induce anthocyanin production, whereas plants Nt#6.1 and Nt#7.5 showed a green phenotype due to the absence or low expression of *AmRosea1*. The unique presence of *AmDelila* was not capable to induce anthocyanin production (plant Nt#6.1). In T0 *N*. *tabacum* plants, there was always the deletion of *MtLAR*, located beside the RB. However, T1 *AmRosea1-AmDelila-MtANR-MtLAR* transgenic plants tend to lose neighbour transgenes located near the LB (*AmRosea1* or *AmDelila*).
(TIF)

S1 Table Primers used in this work. (DOCX)

S2 Table Quantification of PAs in leaf extracts of *Nicotiana benthamiana* agroinfiltrated leaves by the dimethylaminocinnamaldehyde (DMACA) colorimetric reaction. Data represent the mean of three replicates ± the standard error of data for each sample.
(DOCX)

S3 Table Quantification of PAs in leaf extracts of T0 *Nicotiana tabacum* transgenic plants by the dimethylaminocinnamaldehyde (DMACA) colorimetric reaction. Data represent the mean of three replicates ± the standard error of data for each sample. Different letters indicate statistically significant differences, according to the analysis of variance ANOVA (p<0.05).
(DOCX)

S4 Table PA levels and degree of polymerization (mDP) in leaf extracts of T0 *Nicotiana tabacum* transgenic plants estimated by phloroglucinolysis and HPLC/MS. WT: control, non-transformed plant. The results are expressed as mg/g of liophylized leaf material (dry weigh).
(DOCX)

S5 Table PA levels and degree of polymerization (mDP) in leaf extracts of three T1 *Nicotiana tabacum* transgenic plants estimated by phloroglucinolysis and HPLC/MS. WT: control, non-transformed plant. The results are expressed as mg/g of liophylized leaf material (dry weigh).
(DOCX)

## References

[1] Dixon RA, Xie DY, Sharma SB. Proanthocyanidins: a final frontier in flavonoid research? New Phytol. 2005; 165: 9–28 doi: 10.1111/j.1469-8137.2004.01217.x 15720617 10.1111/j.1469-8137.2004.01217.x

[2] Dixon RA. Flavonoids and isoflavonoids: from plant biology to agriculture and neuroscience. Plant Physiol. 2010; 154(2): 453–457 doi: 10.1104/pp.110.161430 20921162 10.1104/pp.110.161430 PMC2948995

[3] Dixon RA, Liu CH, Jun JH. Metabolic engineering of anthocyanins and condensed tannins in plants. Curr. Opin. Biotechnol. 2012; 24: 1–7 22901316 10.1016/j.copbio.2012.07.004

[4] Zhou M, Wei L, Sun Z, Gao L, Meng Y. Tang Y, et al Production and transcriptional regulation of proanthocyanidin biosynthesis in forage legumes. Appl. Microbiol. Biotechnol. 2015; 99: 3797–3806 doi: 10.1007/s00253-015-6533-1 25805345 10.1007/s00253-015-6533-1

[5] Gong ZZ, Yamagishi E, Yamazaki M, Saito K. A constitutively expressed Myc-like gene involved in anthocyanin biosynthesis from *Perilla frutescens*: molecular characterization, heterologous expression in transgenic plants and transactivation in yeast cells. Plant Mol. Biol. 1999; 41: 33–44 10561066 10.1023/a:1006237529040

[6] Ramsay NA, Walker AR, Mooney M, Gray JC. Two basic helix-loop-helix genes (MYC-146 and GL3) from Arabidopsis can activate anthocyanin biosynthesis in a white flowered *Matthiola incana* mutant. Plant Mol. Biol. 2003; 52: 679–688 12956536 10.1023/a:1024852021124

[7] Robbins MP, Paolocci F, Hughes J-W, Turchetti V, Allison G, Arcioni S, et al Sn, a maize bHLH gene, modulates anthocyanin and condensed tannin pathways in *Lotus corniculatus*. J. Exp. Bot. 2003; 54: 239–248 12493851 10.1093/jxb/erg022

[8] Deluc L, Barrieu F, Marchive C, Lauvergeat V, Decendit A, Richard T, et al Characterization of a grapevine R2R3-MYB transcription factor that regulates the phenylpropanoid pathway. Plant Physiol. 2006; 140: 499–511 doi: 10.1104/pp.105.067231 16384897 10.1104/pp.105.067231 PMC1361319

[9] Grotewold E. The genetics and biochemistry of floral pigments. Annu. Rev. Plant Biol. 2006; 57: 761–780 doi: 10.1146/annurev.arplant.57.032905.105248 16669781 10.1146/annurev.arplant.57.032905.105248

[10] Sweeney MT, Thomson MJ, Pfeil BE, McCouch S. Caught redhanded: Rc encodes a basic helix-loop-helix protein conditioning red pericarp in rice. Plant Cell 2006; 18: 283–294 doi: 10.1105/tpc.105.038430 16399804 10.1105/tpc.105.038430 PMC1356539

[11] Li H, Flachowsky H, Fischer TC, Hanke MV, Forkmann G, Treutter D, et al Maize Lc transcription factor enhances biosynthesis of anthocyanins, distinct proanthocyanidins and phenylpropanoids in apple (*Malus domestica* Borkh.). Planta 2007; 226: 1243–1254 doi: 10.1007/s00425-007-0573-4 17618453 10.1007/s00425-007-0573-4

[12] Park KI, Ishikawa N, Morita Y, Choi JD, Hoshino A, Lida S. A bHLH regulatory gene in the common morning glory, *Ipomoea purpurea*, controls anthocyanin biosynthesis in flowers, proanthocyanidin and phytomelanin pigmentation in seeds and trichome formation. Plant J. 2007; 49: 641–654 doi: 10.1111/j.1365-313X.2006.02988.x 17270013 10.1111/j.1365-313X.2006.02988.x

[13] Lloyd AM, Walbot V, Davis RW. Arabidopsis and Nicotiana anthocyanin production activated by maize regulators R and C1. Science 1992; 258: 1773–1775 1465611 10.1126/science.1465611

[14] Xie DY, Sharma SB, Wright E, Wang Z-Y, Dixon RA. Metabolic engineering of proanthocyanidins through co-expression of anthocyanidin reductase and the *PAP1* MYB transcription factor. Plant J. 2006; 45: 895–907 doi: 10.1111/j.1365-313X.2006.02655.x 16507081 10.1111/j.1365-313X.2006.02655.x

[15] Schwinn K, Venail J, Shang YJ, Mackay S, Alm V, Butelli E, et al A small family of MYB-regulatory genes controls floral pigmentation intensity and patterning in the genus Antirrhinum. Plant Cell 2006; 18: 831–851 doi: 10.1105/tpc.105.039255 16531495 10.1105/tpc.105.039255 PMC1425845

[16] Orzáez D, Medina A, Torre S, Fernández-Moreno JP, Rambla JL, Fernández-Del-Carmen A, et al A visual reporter system for virus-induced gene silencing in tomato fruit based on anthocyanin accumulation. *Plant Physiol*. 2009; 150(3): 1122–34. doi: 10.1104/pp.109.139006 19429602 10.1104/pp.109.139006 PMC2705029

[17] Butelli E, Titta L, Giorgio M, Mock H-P, Matros A, Peterek S, et al Enrichment of tomato fruit with health-promoting anthocyanins by expression of select TFs. Nat Biotechnol. 2008; 26:1301–1308 doi: 10.1038/nbt.1506 18953354 10.1038/nbt.1506

[18] Tanner GJ, Francki KT, Abrahams S, Watson JM, Larkin PJ, Ashton AR. Proanthocyanidin biosynthesis in plants. Purification of legume leucoanthocyanidin reductase and molecular cloning of its cDNA. J. Biol. Chem. 2003; 278: 31647–31656 doi: 10.1074/jbc.M302783200 12788945 10.1074/jbc.M302783200

[19] Xie DY, Sharma SB, Paiva NL, Ferreira D, Dixon RA. Role of anthocyanidin reductase, encoded by *BANYULS* in plant flavonoid biosynthesis. Science 2003; 299: 396–399 doi: 10.1126/science.1078540 12532018 10.1126/science.1078540

[20] Xie DY, Sharma SB, Dixon RA. Anthocyanidin reductases from *Medicago truncatula* and *Arabidopsis thaliana*. Arch. Biochem. Biophys. 2004; 422: 91–102 14725861 10.1016/j.abb.2003.12.011

[21] Pang Y, Peel GJ, Wright E, Wang Z, Dixon RA. Early steps in proanthocyanidin biosynthesis in the model legume *Medicago truncatula*. Plant Physiol. 2007; 145: 601–615 doi: 10.1104/pp.107.107326 17885080 10.1104/pp.107.107326 PMC2048810

[22] Capell T and Christou P. Progress in plant metabolic engineering. Curr. Opin. Biotechnol. 2004; 15: 148–154 doi: 10.1016/j.copbio.2004.01.009 15081054 10.1016/j.copbio.2004.01.009

[23] Halpin C. Gene stacking in transgenic plants-the challenge for 21^st^ century plant biotechnology. Plant Biotechnol. J. 2005; 3: 141–155 doi: 10.1111/j.1467-7652.2004.00113.x 17173615 10.1111/j.1467-7652.2004.00113.x

[24] Dafny-Yelin M and Tzfira T. Delivery of multiple transgenes to plant cells. Plant Physiol. 2007; 145: 1118–1128 doi: 10.1104/pp.107.106104 18056862 10.1104/pp.107.106104 PMC2151730

[25] Ma JK, Hiatt A, Hein M, Vine ND, Wang F, Stabila P, et al Generation and assembly of secretory antibodies in plants. Science 1995; 268: 716–719 7732380 10.1126/science.7732380

[26] Datta K, Baisakh N, Maung Thet K, Tu J, Datta SK. Pyramiding transgenes for multiple resistance in rice against bacterial blight, yellow stem borer and sheath blight. Theor. Appl. Genet. 2002; 106: 1–8 doi: 10.1007/s00122-002-1014-1 12582865 10.1007/s00122-002-1014-1

[27] Lapierre C, Pollet B, Petit-Conil M, Toval G, Romero J, Pilate G, et al Structural alterations of lignins in transgenic poplars with depressed cinnamyl alcohol dehydrogenase or caffeic acid methyltransferase activity have an opposite impact on the efficiency of industrial kraft pulping. Plant Physiol. 1999; 119: 153–163 9880356 10.1104/pp.119.1.153 PMC32214

[28] Jobling SA, Westcott RJ, Tayal A, Jeffcoat R, Schwall GP. Production of a freeze–thaw-stable potato starch by antisense inhibition of three starch synthase genes. Nat. Biotechnol. 2002; 20: 295–299 doi: 10.1038/nbt0302-295 11875432 10.1038/nbt0302-295

[29] Qi B, Fraser T, Mugford S, Dobson G, Sayanova O, Butler J, et al Production of very long chain polyunsaturated omega-3 and omega-6 fatty acids in plants. Nat. Biotechnol. 2004; 22: 739–745 doi: 10.1038/nbt972 15146198 10.1038/nbt972

[30] Halpin C, Barakate A, Askari B, Abbott J, Ryan MD. Enabling technologies for manipulating multiple genes on complex pathways. Plant Mol. Biol. 2001; 47: 295–310 11554478

[31] Cheo DL, Titus SA, Byrd DR, Hartley JL, Temple GF, Brasch MA. Concerted assembly and cloning of multiple DNA segments using in vitro site-specific recombination: functional analysis of multi-segment expression clones. Genome Res. 2004; 14: 2111–2120 doi: 10.1101/gr.2512204 15489333 10.1101/gr.2512204 PMC528927

[32] Magnani E, Bartling L, Hake S. From Gateway to MultiSite Gateway in one recombination event. BMC Mol. Biol. 2006; 7: 46 doi: 10.1186/1471-2199-7-46 17150110 10.1186/1471-2199-7-46 PMC1702363

[33] Engler C, Kandzia R, Marillonnet S. A one pot, one step, precision cloning method with high throughput capability. PLoS One 2008; 3: e3647 doi: 10.1371/journal.pone.0003647 18985154 10.1371/journal.pone.0003647 PMC2574415

[34] Sarrion-Perdigones A, Falconi EE, Zandalinas SI, Juarez P, Fernandez-del-Carmen A, Granell A, et al GoldenBraid: An Iterative Cloning System for Standardized Assembly of Reusable Genetic Modules. PLoS ONE 2011; 6 (7) e21622 doi: 10.1371/journal.pone.0021622 21750718 10.1371/journal.pone.0021622 PMC3131274

[35] Sarrion-Perdigones A, Vázquez-Vilar M, Palací J, Castelijns B, Forment J, Ziarsolo P, et al GOLDENBRAID 2.0: A comprehensive DNA assembly framework for Plant Synthetic Biology. Plant Physiol. 2013; 162(3): 1618–31 doi: 10.1104/pp.113.217661 23669743 10.1104/pp.113.217661 PMC3707536

[36] Sarrion-Perdigones A, Palaci J, Granell A, Orzaez D. Design and Construction of Multigenic Constructs for Plant Biotechnology Using the GoldenBraid Cloning Strategy. DNA Cloning and Assembly Methods, Methods in Molecular Biology, 2014; vol. 1116, doi: 10.1007/978-1-62703-764-8_10 24395362 10.1007/978-1-62703-764-8_10

[37] Vafaee Y, Staniek A, Mancheno-Solano M, Warzecha H. A Modular Cloning Toolbox for the Generation of Chloroplast Transformation Vectors. Isalan M, ed. PLoS ONE 2014; 9(10):e110222 doi: 10.1371/journal.pone.0110222 25302695 10.1371/journal.pone.0110222 PMC4193872

[38] Polturak G, Breitel D, Grossman N, Sarrion-Perdigones A, Weithorn E, Pliner M, et al Elucidation of the first committed step in betalain biosynthesis enables the heterologous engineering of betalain pigments in plants. New Phytol. 2016; 210 (1): 269–83 doi: 10.1111/nph.13796 26683006 10.1111/nph.13796

[39] Hewitt EJ. Sand and water culture methods used in the study of plant nutrition. Farnham Royal, UK: Commonwealth Agricultural Bureau, 1966

[40] Vázquez-Vilar M, Bernabé-Orts JM, Fernandez-del-Carmen A, Ziarsolo P, Blanca J, Granell A, et al A modular toolbox for gRNA–Cas9 genome engineering in plants based on the GoldenBraid standard. Plant Methods 2016; 12:10 doi: 10.1186/s13007-016-0101-2 26839579 10.1186/s13007-016-0101-2 PMC4736081

[41] Wieland WH, Lammers A, Schots A, Orzaez DV. Plant expression of chicken secretory antibodies derived from combinatorial libraries. J. Biotechnol 2006; 122: 382–391 doi: 10.1016/j.jbiotec.2005.12.020 16448714 10.1016/j.jbiotec.2005.12.020

[42] Horsch RB, Fraley RT, Rogers SG, Sanders PR, Lloyd A, Hoffmann N. Inheritance of functional foreign genes in plants. Science 1984; 223: 496–498 doi: 10.1126/science.223.4635.496 17781445 10.1126/science.223.4635.496

[43] Fisher DK and Guiltinan J. Rapid, efficient production of homozygous transgenic tobacco plants with *Agrobacterium tumefaciens*: a seed to seed protocol. *Plant Mol Biol Rep*. 1995; 13: 278–289

[44] Modolo L, Blount JW, Achnine L, Naoumkina M, Wang X, Dixon RA. A functional genomics approach to (iso)flavonoid glycosylation in the model legume *Medicago truncatula*. Plant Mol. Biol. 2007; 64: 499*–*518 doi: 10.1007/s11103-007-9167-6 17437063 10.1007/s11103-007-9167-6

[45] Pang Y, Abeysinghe ISB, He J, He X, Huhman D, Mewan KM, et al Functional characterization of proanthocyanidin pathway enzymes from tea and their application for metabolic engineering. Plant Physiol. 2013, 161: 1103–1116 doi: 10.1104/pp.112.212050 23288883 10.1104/pp.112.212050 PMC3585583

[46] de Pascual-Teresa S, Treutter D, Rivas-Gonzalo JC, Santos-Buelga C. Analysis of Flavanols in Beverages by High-Performance Liquid Chromatography with Chemical Reaction Detection. J. Agric. Food Chem. 1998; 46: 4209–4213

[47] Delcour JA, de Varebeke DJ. A new colourimetric assay for flavanoids in pilsner beers. J. Inst. Brew. 1985; 91: 37–40

[48] Kennedy JA, and Jones GP. Analysis of proanthocyanidins cleavage products following acid-catalysis in the presence of an excess phloroglucinol. J. Agric. Food Chem. 2001; 49: 1740–1746 11308320 10.1021/jf001030o

[49] Buendía B, Gil MI, Tudela J A, Gady AL, Medina JJ, Soria C, et al HPLC–MS Analysis of proanthocyanidin oligomers and other phenolics in 15 strawberry cultivars. J. Agric. Food Chem. 2010; 58: 3916–3926 doi: 10.1021/jf9030597 20038100 10.1021/jf9030597

[50] Vallejo F, Marín JG, Tomás-Barberán FA. Phenolic compound content of fresh and dried figs (*Ficus carica* L.). Food Chem. 2012; 130: 485–492

[51] Kitamura S. Transport of Flavonoids: From Cytosolic Synthesis to Vacuolar Accumulation In *The Science of Flavonoids*: Springer New York, 2006; pp. 123–146

[52] Laitinen RAE, Ainasoja M, Broholm SK, Teeri TH, Elomaa P. Identification of target genes for a MYB-type anthocyanin regulator in *Gerbera hybrida*. J. Exp. Botany 2008; 59: 3691–3703 18725377 10.1093/jxb/ern216 PMC2561154

[53] Du H, Huang Y, Tang Y. Genetic and metabolic engineering of isoflavonoid biosynthesis. Appl. Microbiol. Biotechnol. 2010; 86, 1293–1312 doi: 10.1007/s00253-010-2512-8 20309543 10.1007/s00253-010-2512-8

[54] Zhang Y-J, Gan R-Y, Li S, Zhou Y, Li A-N, Xu D-P, et al Antioxidant Phytochemicals for the Prevention and Treatment of Chronic Diseases. Molecules 2015; 20: 21138–21156 doi: 10.3390/molecules201219753 26633317 10.3390/molecules201219753 PMC6331972

[55] Nakatsuka T, Haruta KS, Pitaksutheepong C, Abe Y, Kakizaki Y, Yamamoto K, et al Identification and characterization of R2R3-MYB and bHLH transcription factors regulating anthocyanin biosynthesis in gentian flowers. Plant Cell Physiol. 2008; 49: 1818–1829 doi: 10.1093/pcp/pcn163 18974195 10.1093/pcp/pcn163

[56] Albert NW, Lewis DH, Zhang H, Schwinn KE, Jameson PE, Davies KM. Members of an R2R3-MYB transcription factor family in Petunia are developmentally and environmentally regulated to control complex floral and vegetative pigmentation patterning. Plant J. 2011; 65: 771–784 doi: 10.1111/j.1365-313X.2010.04465.x 21235651 10.1111/j.1365-313X.2010.04465.x

[57] Albert NW, Davies KM, Lewis DH, Zhang H, Montefiori M, Brendolise C, et al A conserved network of transcriptional activators and repressors regulates anthocyanin pigmentation in eudicots. Plant Cell 2014; 26: 962–980 doi: 10.1105/tpc.113.122069 24642943 10.1105/tpc.113.122069 PMC4001404

[58] Verdier J, Zhao J, Torres-Jerez I, Ge S, Liu CH, He X, et al *MtPAR* MYB transcription factor acts as an on switch for proanthocyanidin biosynthesis in *Medicago truncatula*. Proc Natl Acad Sci U S A 2012; 109(5): 1766–1771 doi: 10.1073/pnas.1120916109 22307644 10.1073/pnas.1120916109 PMC3277187

[59] Binns AN and Thomashow MF. Cell Biology of Agrobacterium Infection and Transformation of Plants. Ann. Rev. Microbiol. 1988; 42: 575–606

[60] Zambryski P. Basic processes underlying Agrobacterium-mediated DNA transfer to plant cells. Annu Rev Genet. 1988; 22: 1–30 doi: 10.1146/annurev.ge.22.120188.000245 3071244 10.1146/annurev.ge.22.120188.000245

[61] Kim H, Seok Park B, Park YD, Jin YM. Pollen ablation of transgenic tobacco plants by expression of the diphtheria toxin A-chain gene under the control of a putative pectin esterase promoter from Chinese cabbage. Mol Cells 1998; 8: 310–317 9666468

[62] Ch Liu., Wang X, Shulaev V, Dixon RA. A role for leucoanthocyanidin reductase in the extension of proanthocyanidins. Nature Plants 2016; 2: 1–7 10.1038/nplants.2016.182 27869786

[63] Li P, Chen B, Zhang G, Chen L, Dong Q, Wen J, et al Regulation of anthocyanin and proanthocyanidin biosynthesis by *Medicago truncatula* bHLH transcription factor *MtTT8*. New Phytologist 2016; 210(3): 905–21 doi: 10.1111/nph.13816 26725247 10.1111/nph.13816

[64] Paolocci F, Robbins MP, Madeo L, Arcioni S, Martens S, Damiani F. Ectopic expression of basic helix-loop-helix gene transactivates parallel pathways of proanthocyanidin biosynthesis: structure, expression analysis and genetic control of leucoanthocyanidin 4-reductase and anthocyanidin reductase genes in *Lotus corniculatus*. Plant Physiol. 2007; 143: 504–516 doi: 10.1104/pp.106.090886 17098849 10.1104/pp.106.090886 PMC1761954

[65] Liu Y, Shi Z, Maximova S, Payne MJ, Guiltinan MJ. Proanthocyanidin synthesis in *Theobroma cacao*: genes encoding anthocyanidin synthase, anthocyanidin reductase, and leucoanthocyanidin reductase. BMC Plant Biol. 2013; 13: 202 doi: 10.1186/1471-2229-13-202 24308601 10.1186/1471-2229-13-202 PMC4233638

[66] Li YG, Tanner G, Larkin P. The DMACA-HCl. Protocol and the threshold proanthocyanidin content for bloat safety in forage legumes. J. Sci. Food Agric. 1996; 70: 89–101

[67] Hall J, Walker I, Majak W. Evaluation of two supplements for the prevention of alfalfa bloat. Can. Vet. J. 1994; 35: 702–705 7866960 PMC1686822

[68] Waghorn GC. Beneficial effects of low concentrations of condensed tannins in forages fed to ruminants In Akin DE, Ljungdahl LLG, Wilson JR, Harris PJ, eds, Microbial and Plant Opportunities to Improve Lignocellulose Utilization by Ruminants. Elsevier, New York, 1990; pp 137–147

[69] Lees GL. Condensed tannins in some forage legumes: their role in the prevention of ruminant pasture bloat. Basic Life Sci. 1992; 59: 915–934 1417702 10.1007/978-1-4615-3476-1_55

[70] Niezen KE, Waghorn TS, Charleston WAC, Waghorn GC. Growth and gastrointestinal nematode parasitism in lambs grazing either Lucerne (*Medicago sativa*) or sulla (*Hedysarum coronarium*) which contains condensed tannins. J Agric. Sci. 1995; 125: 281–289

[71] Neizen JH, Robertson HA, Waghorn GC, Charleston WAC. Production, faecal egg counts and worm burdens of ewe lambs which grazed six contrasting forages. Veterin. Parasitol. 1998; 80: 15–27 10.1016/s0304-4017(98)00202-7 9877067

[72] Aerts RJ, Barry TN, McNabb WC. Polyphenols and agriculture: beneficial effects of proanthocyanidins in forages. Agric Ecosyst Environ. 1999; 75: 1–12

[73] Muir AD, Gruber MY, Hinks CF, Lees GL, Onyilagha J, Hallett R, et al The effects of condensed tannin in the diets of major crop insects In Gross GG, Hemingway RW, Yoshida T, eds, Plant Polyphenols 2: Chemistry, Biology, Pharmacology, Ecology. Plenum Press, New York, 1999; pp 867–881

[74] McMahon LR, McAllister TA, Berg BP, Majak W, Acharya SN, Popp JD, et al A review of the effects of forage condensed tannins on ruminal fermentation and bloat in grazing cattle. Can. J. Plant Sci. 2000; 80: 469–485

[75] Barry TN and McNabb WC. The implications of condensed tannins on the nutritive value of temperate forages fed to ruminants. Br. J. Nutr. 1999; 81: 263–272 10999013

[76] Peel GJ, Pang Y, Modolo LV, Dixon RA. The *LAP1* MYB transcription factor orchestrates anthocyanidin biosynthesis and glycosylation in Medicago. Plant J. 2009; 59: 136–149 doi: 10.1111/j.1365-313X.2009.03885.x 19368693 10.1111/j.1365-313X.2009.03885.x

[77] Baudry A, Heim MA, Dubreucq B, Caboche M, Weisshaar B, Lepiniec L. TT2, TT8, and TTG1 synergistically specify the expression of *BANYULS* and proanthocyanidin biosynthesis in *Arabidopsis thaliana*. Plant J. 2004; 39: 366–380 doi: 10.1111/j.1365-313X.2004.02138.x 15255866 10.1111/j.1365-313X.2004.02138.x

[78] Ch Liu, Jun JH, Dixon RA. *MYB5* and *MYB14* play pivotal roles in seed coat polymer biosynthesis in *Medicago truncatula*. Plant Physiol. 2014; 165: 1424–1439 doi: 10.1104/pp.114.241877 24948832 10.1104/pp.114.241877 PMC4119029

