# Supplementary figures and images for: Metabolic engineering to simultaneously activate anthocyanin and proanthocyanidin biosynthetic pathways in Nicotiana spp
Source: PLoS One. 2017 Sep 13;12(9):e0184839. doi: 10.1371/journal.pone.0184839 (PMC5597232; doi:10.1371/journal.pone.0184839)

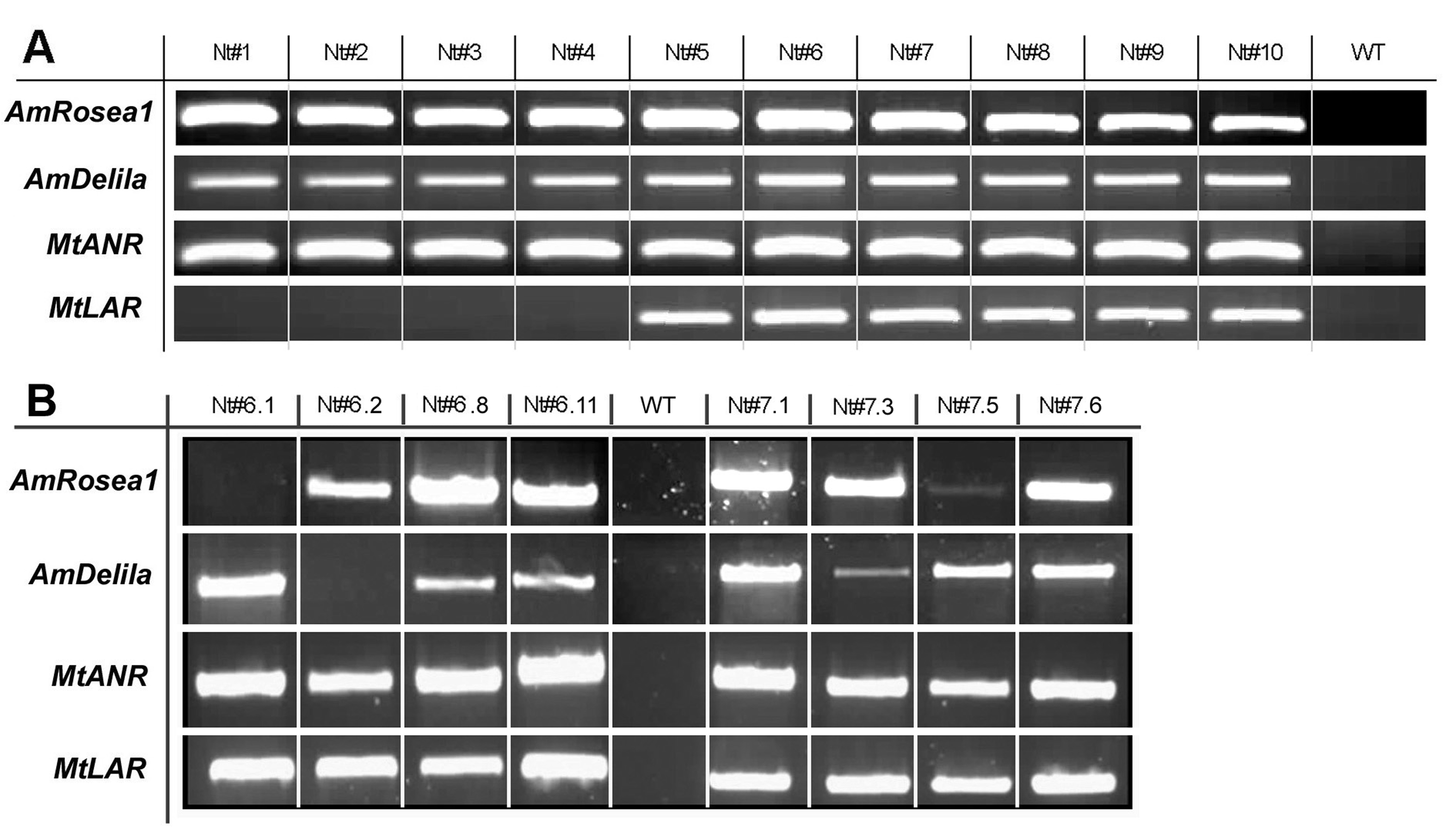

Supplement: S1 Fig — (A) Detection by PCR of the presence of the AmRosea1, AmDelila, MtANR and MtLAR transgenes in T0 N. tabacum transgenic plants. 6 out of 10 plants incorporated the complete full set of transgenes. Plants Nt#1 to Nt#4 lacked the MtLAR transgen. (B) Detection by PCR of the presence of the AmRosea1, AmDelila, MtANR and MtLAR transgenes in some plants of the T1 lineage of N. tabacum Nt#6 and Nt#7 transgenic plants. About the 60% of the T1 plants incorporated the full set of transgenes. The unique expression of AmRosea1 in the Nt#6.2 plant was sufficient to induce anthocyanin production, whereas plants Nt#6.1 and Nt#7.5 showed a green phenotype due to the absence or low expression of AmRosea1. The unique presence of AmDelila was not capable to induce anthocyanin production (plant Nt#6.1). In T0 N. tabacum plants, there was always the deletion of MtLAR, located beside the RB. However, T1 AmRosea1-AmDelila-MtANR-MtLAR transgenic plants tend to lose neighbour transgenes located near the LB (AmRosea1 or AmDelila). (TIF) [file pone.0184839.s001.tif]
