# Supplementary material for: Metabolic engineering to simultaneously activate anthocyanin and proanthocyanidin biosynthetic pathways in Nicotiana spp
Source: PLoS One. 2017 Sep 13;12(9):e0184839. doi: 10.1371/journal.pone.0184839 (PMC5597232; doi:10.1371/journal.pone.0184839)
Supplement: S1 Table — (DOCX) [file pone.0184839.s002.docx]

| **Gene** | **Sequence primer 5´-3´** |
| --- | --- |
| ***MtANR-F1*** | GCGCCGTCTCACTCGAATGGCTAGTATCAAACAAATAGAAATAG |
| ***MtANR-R1*** | GCGCCGTCTCATCCCTAACAGTAGTATTAACAG |
| ***MtANR-F2*** | GCGCCGTCTCAGGGACCTAGATAGTGCAAACAAAAC |
| ***MtANR-R2*** | GCGCCGTCTCTCGCTCGAAGCTCACTTGATCCCCTGAGTCTTC |
| ***MtLAR-F1*** | GCGCCGTCTCACTCGAATGGCACCATCATCATCACCAACC |
| ***MtLAR-R1*** | GCGCCGTCTCACACGATTGCTCTAGGAATTTTTC |
| ***MtLAR-F2*** | GCGCCGTCTCACGTGTCTGAAGACGATCTTCTAG |
| ***MtLAR-R2*** | GCGCCGTCTCGCTCGAAGCTCAACAGGAAGCTGTGATTGGC |
| ***NbActin DIR*** | GGATGTGAAGGAGAAGTTGG |
| ***NbActin REV*** | GCTCATCCTATCAGCAATGC |
| ***DELILA DIR*** | GAGGAAGCAACTTGCTATTGCTG |
| ***DELILA REV*** | CATTGATGCCTTCTGCCAAATTCG |
| ***ROSEA 244 DIR*** | Ggtaacaaatggtcgctgattgc |
| ***ROSEA 618 REV*** | ccatcctccgttgtttctagc |
| ***MtANR 39 DIR*** | GCATGTGTGATAGGTGGAC |
| ***MtANR 419 REV*** | GACCAGTCCCTTCGAGTTCG |
| ***MtLAR 39 DIR*** | GGTCGAGTCCTAATTGTTGG |
| ***MtLAR 356 REV*** | CGTGACCAAATTCTGAAGGC |
| ***qRT-DELILA DIR*** | TACGGACAAGATTGGAGCCG |
| ***qRT-DELILA REV*** | GGAATCTTTCAATCGACCTCTG |
| ***qRT-ROSEA1 DIR*** | GACTAATATCGTAAGACCCCGAGC |
| ***qRT-ROSEA1 REV*** | GTAACGTGCAATCCGGTGAAGG |
| ***qRT-MtANR DIR*** | CAACTTCTGGTCGATACATTTGC |
| ***qRT-MtANR REV*** | CGGGAACACTGGTATTGTGAG |
| ***qRT-MtLAR DIR*** | CCACCGTTGGATCAATTGCAC |
| ***qRT-MtLAR REV*** | GTACGCTTTGACATTGCCATG |
| ***Semi-NbCHS DIR*** | ATAGGTTCTGATCCAATTCCAG |
| ***Semi-NbCHS REV*** | CTGTGGAGAACAACAGTCTCAACT |
| ***Semi-NtCHI DIR*** | ACGGGTAAGCAATACTCAGAGAAG |
| ***Semi-NtCHI REV*** | TAGACTCCAATTTCTGGAATGGT |
| ***Semi-NtF3H DIR*** | TATCCAATTCGGGCTAGAGACTAC |
| ***Semi-NtF3H REV*** | GGGTACACTATAGCTTCTGGTGCT |
| ***Semi-NbDFR1 DIR*** | CCTAGCTTAATCACTGCACTTTCA |
| ***Semi-NbDFR1 REV*** | ATTGGTTGACTTTCCTGTTCCATT |
| ***Semi-NtANS DIR*** | CTGGCCTAAAATCCCTACTGACTA |
| ***Semi-NtANS REV*** | TCTCTTTATTCACAACCCCTCTGT |

**S1 Table.**
