# Supplementary material for: Metabolic engineering to simultaneously activate anthocyanin and proanthocyanidin biosynthetic pathways in Nicotiana spp
Source: PLoS One. 2017 Sep 13;12(9):e0184839. doi: 10.1371/journal.pone.0184839 (PMC5597232; doi:10.1371/journal.pone.0184839)
Supplement: S2 Table — Data represent the mean of three replicates ± the standard error of data for each sample. (DOCX) [file pone.0184839.s003.docx]

| **Samples** | **nmol (-)-epicatechin/g FW** | **nmol (-)-catechin/g FW** |
| --- | --- | --- |
| **WT** | 2.04 ± 1.33 | 1.86 ± 1.22 |
| ***AmROSEA1-AmDELILA-MtANR-MtLAR*** | 228.51 ± 24.17 | 208.56 ± 31.19 |

**S2 Table.**
