# Supplementary material for: Metabolic engineering to simultaneously activate anthocyanin and proanthocyanidin biosynthetic pathways in Nicotiana spp
Source: PLoS One. 2017 Sep 13;12(9):e0184839. doi: 10.1371/journal.pone.0184839 (PMC5597232; doi:10.1371/journal.pone.0184839)
Supplement: S3 Table — Data represent the mean of three replicates ± the standard error of data for each sample. Different letters indicate statistically significant differences, according to the analysis of variance ANOVA (p<0.05). (DOCX) [file pone.0184839.s004.docx]

| **Samples** | **nmol (-)-epicatechin/g FW** | **nmol (-)-catechin/g FW** |
| --- | --- | --- |
| **WT** | 16.90 ± 0.42 a | 15.42 ± 0.38 a |
| ***AmROSEA1-AmDELILA-MtANR-MtLAR* (Nt#7)** | 633 ± 27,5 c | 577.79 ± 25.09 c |
| ***AmROSEA1-AmDELILA-MtANR-MtLAR* (Nt#6)** | 86.56 ± 4.91 b | 79 ± 4.48 b |

**S3 Table.**
