# Supplementary material for: Metabolic engineering to simultaneously activate anthocyanin and proanthocyanidin biosynthetic pathways in Nicotiana spp
Source: PLoS One. 2017 Sep 13;12(9):e0184839. doi: 10.1371/journal.pone.0184839 (PMC5597232; doi:10.1371/journal.pone.0184839)
Supplement: S4 Table — WT: control, non-transformed plant. The results are expressed as mg/g of liophylized leaf material (dry weigh). (DOCX) [file pone.0184839.s005.docx]

|  | **Epicatechin** | **Catechin** | **Epicatechin**  **aduct** | **Total**  **procyanidins** | **mDP** |
| --- | --- | --- | --- | --- | --- |
| **WT** | -- | -- | -- | -- | -- |
| **Nt#7** | 1.42 ± 0.32 | 0.57 ± 0.14 | 1.39 ± 0.65 | 3.48 ± 1.09 | 1.76 ± 0.56 |
| **Nt#6** | 0.02 ± 0.00 | 0.06 ± 0.01 | 0.09 ± 0.01 | 0.15 ± 0.05 | 2.32 ± 0.12 |
| **Nt#5** | -- | 0.10 ± 0.01 | -- | 0.10 ± 0.01 | -- |

**S4 Table.**
