# Supplementary material for: Metabolic engineering to simultaneously activate anthocyanin and proanthocyanidin biosynthetic pathways in Nicotiana spp
Source: PLoS One. 2017 Sep 13;12(9):e0184839. doi: 10.1371/journal.pone.0184839 (PMC5597232; doi:10.1371/journal.pone.0184839)
Supplement: S5 Table — WT: control, non-transformed plant. The results are expressed as mg/g of liophylized leaf material (dry weigh). (DOCX) [file pone.0184839.s006.docx]

|  | **Epicatechin** | **Catechin** | **Epicatechin**  **aduct** | **Total**  **procyanidins** | **mDP** |
| --- | --- | --- | --- | --- | --- |
| **WT** | -- | -- | -- | -- | -- |
| **Nt#6.8** | 0.273 ±0.004 | 0.268 ± 0.005 | 0.657 ± 0.08 | 1.197 ± 0.08 | 2.216 ± 0.015 |
| **Nt#6.11** | 0.642 ± 0.22 | 0.186 ± 0.1 | 1.089 ± 0.21 | 1.916 ± 0.53 | 2.366 ± 0.26 |
| **Nt#7.6** | 0.612 ±0.38 | 0.231 ±0.16 | 1.530 ±0.64 | 2.372 ± 0.73 | 2.820 ± 0.14 |

**S5 Table.**
